# Evolutionary games of condensates in coupled birth–death processes

**DOI:** 10.1038/ncomms7977

**Published:** 2015-04-24

**Authors:** Johannes Knebel, Markus F. Weber, Torben Krüger, Erwin Frey

**Affiliations:** 1 Arnold Sommerfeld Center for Theoretical Physics and Center for NanoScience, Department of Physics, Ludwig-Maximilians-Universität München, Theresienstraße 37, 80333 München, Germany; 2 IST Austria, Am Campus 1, 3400 Klosterneuburg, Austria

## Abstract

Condensation phenomena arise through a collective behaviour of particles. They are observed in both classical and quantum systems, ranging from the formation of traffic jams in mass transport models to the macroscopic occupation of the energetic ground state in ultra-cold bosonic gases (Bose–Einstein condensation). Recently, it has been shown that a driven and dissipative system of bosons may form multiple condensates. Which states become the condensates has, however, remained elusive thus far. The dynamics of this condensation are described by coupled birth–death processes, which also occur in evolutionary game theory. Here we apply concepts from evolutionary game theory to explain the formation of multiple condensates in such driven-dissipative bosonic systems. We show that the vanishing of relative entropy production determines their selection. The condensation proceeds exponentially fast, but the system never comes to rest. Instead, the occupation numbers of condensates may oscillate, as we demonstrate for a rock–paper–scissors game of condensates.

## Abstract (web summary)

A driven-dissipative system of non-interacting bosons may form multiple condensates—a dynamics described by birth–death processes that also occur in evolutionary game theory. Here, the authors apply game theory to show how the vanishing of relative entropy production governs condensate selection.

## Graphical abstract

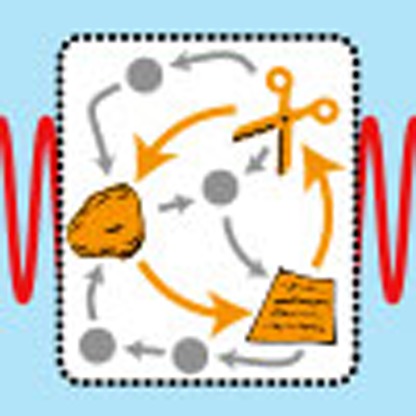

Condensation phenomena occur in a broad range of contexts in both classical and quantum systems. Networks such as the World Wide Web or the citation network perpetually grow by the addition of nodes or links and they evolve by rewiring. Over time, a finite fraction of the links of a network may be attached to particular nodes. These nodes become hubs and thereby dominate the dynamics of the whole network; they become condensate nodes^1^,^2^,^3^. Condensation also occurs in models for the jamming of traffic^4^,^5^,^6^,^7^ and in related mass transport models in which particles hop between sites on a lattice^3^,^8^,^9^. A condensate forms when a finite fraction of all particles aggregates into a cluster that dominates the total particle flow. Bose–Einstein condensation, on the other hand, is a quintessentially quantum mechanical phenomenon. When an equilibrated, dilute gas of bosonic particles is cooled to a temperature near absolute zero, a finite fraction of bosons may condense into the energetic ground state^10^,^11^,^12^. Long-range phase coherence builds up and quantum physics becomes manifest on the macroscopic scale^13^,^14^.

In both the classical and the quantum mechanical context, condensation occurs when one or multiple states become macroscopically occupied (they become condensates), whereas the other states become depleted^15^,^16^. However, the physical origins of condensation in the above examples differ from each other. Why and how condensation arises in a particular system remains a topic of general interest and vivid research.

Here we study condensation in two systems from different fields of research: incoherently driven-dissipative systems of non-interacting bosons and evolutionary games of competing agents. As we show below, the physical principle of vanishing entropy production governs the formation of condensates in both of these systems. The entities that constitute the respective system shall be called particles. They may be quantum or classical particles (bosons or agents). The dynamics of these particles eventually lead to the condensation into particular states (quantum states or strategies). Before describing the above two systems, we now introduce the mathematical framework of our study.

On an abstract level, we consider a system of *S* (non-degenerate) states 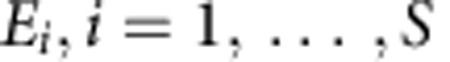, each of which is occupied by *N*_*i*_≥0 indistinguishable particles, see Fig. 1a. The configuration of the system at time *t* is fully characterized by the occupation numbers 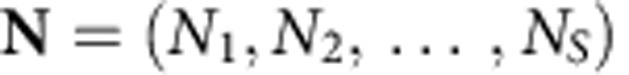. This configuration changes continuously in time due to the transition of particles between states. The total number of particles in this coupled birth–death process is conserved 
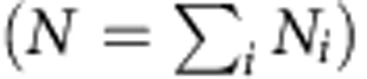
. We are interested in the probability *P*(**N**, *t*) of finding the system in configuration **N** at time *t*. The temporal evolution of the probability distribution *P*(**N**, *t*) is governed by the classical master equation^17^,^18^:


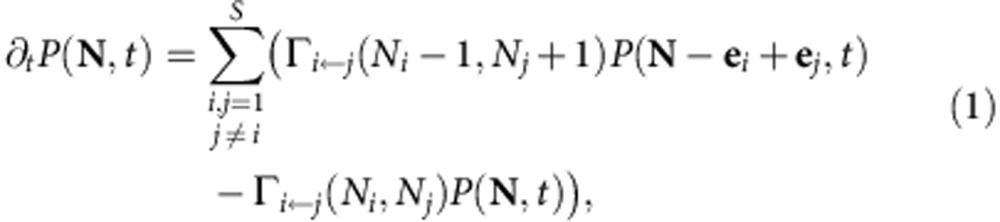


where 
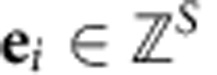
 denotes the unit vector in direction *i* (equal to 1 at index *i*, otherwise 0). The rate for the transition of particles from state *E*_*j*_ to *E*_*i*_ depends linearly on the number of particles in the departure and in the arrival state:


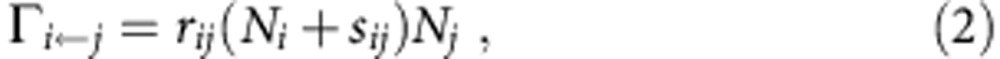


with rate constant *r*_*ij*_≥0 and constant *s*_*ij*_≥0.

Condensation in this framework is understood as the macroscopic occupation of one or multiple states^15^,^16^: We consider a state *E*_*i*_ as a condensate when the long-time average of the number of particles in this state scales linearly with the system size (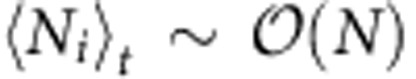 for large *t*). Hence, a condensate harbours a finite fraction of the total number of particles for large systems (
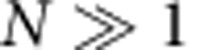
). We refer to a state as depleted when its average occupation number scales less than linearly with the system size. Therefore, the fraction of particles in a depleted state vanishes in the limit of large systems.

Depending on the values of the rate constants *r*_*ij*_, numerical simulation of equations (1) with rates (2), 6 reveals that all states, multiple states or only one state become condensates when the particle density *N*/*S* is large enough to detect condensation^19^. Thus far, various questions about condensation have remained elusive for the coupled birth–death process defined by equation (1), 6: Which of the states become condensates? How does this selection of condensates proceed? Is it possible to construct systems that condense into a specific set of condensates?

In the following, we answer these questions by illuminating the physical principle that governs the formation of multiple condensates on the leading-order timescale. We show that the vanishing of relative entropy production determines the selection of condensates (see equations (3) and (4) below). We elaborate how condensate selection is determined by the rate constants *r*_*ij*_. The condensation proceeds exponentially fast into a dynamic, metastable steady state within which the occupation numbers of condensates may oscillate. By applying our general results to systems with many states, we show that the interplay between critical properties of such networks of states^20^ and dynamically stable network motifs^21^ determines the selection of condensates. The results of our analysis apply to any system whose dynamics are described by the coupled birth–death processes (1) with rates (2). Before proceeding to the mathematical and numerical analysis of condensation in these processes, we now give a brief overview of such systems.

## Results

### Non-interacting bosons in driven-dissipative systems

The classical master equation (1) has recently been derived by Vorberg *et al.*^19^ in the study of bosonic systems that are dissipative and driven by external sources. For a system of non-interacting bosons that is weakly coupled to a reservoir and driven by an external time-periodic force (a so-called Floquet system)^22^,^23^,^24^, one can eliminate the reservoir degrees of freedom (Born and Markov approximation)^25^,^26^ and the density matrix of the system becomes diagonal (see the Supplement of the work of Vorberg *et al.*^19^). The effective dynamics of the bosons become incoherent and are captured on a macroscopic level in terms of the coupled birth–death processes (1) with rates Γ_*i*←*j*_=*r*_*ij*_(*N*_*i*_+1)*N*_*j*_ (that is all *s*_*ij*_=1 in the rates (2)). These non-equilibrium set-ups may not only lead the bosons into a single, but also into multiple condensates^19^.

For the incoherently driven-dissipative systems described above, the state *E*_*i*_ denotes a time-dependent Floquet state^22^,^23^,^24^. The total rate Γ_*i*←*j*_ for the transition of a boson from state *E*_*j*_ to *E*_*i*_ depends linearly on the number of bosons in the departure state (*N*_*j*_) and the arrival state (*N*_*i*_+1). The latter factor stems from the indistinguishability of bosons and reflects their tendency to congregate. Although we refer to equation (1) as a classical master equation and coherence does not build up, the quantum statistics of bosons is still encoded in the functional form of Γ_*i*←*j*_. The rate constant *r*_*ij*_ is determined by the microscopic properties of the system and the reservoir.

Condensation in the above set-up is to be distinguished from Bose–Einstein condensation. Typically, studies on Bose–Einstein condensation focus on the existence of long-range phase coherence in thermal equilibrium^10^,^11^,^12^,^13^,^14^,^15^, its kinetic formation^13^,^14^,^27^,^28^,^29^,^30^,^31^,^32^ and the fragmentation of a coherent condensate into multiple condensates (for example, when the equilibrium ground state is degenerate)^13^,^16^. In contrast, the classical birth–death processes (1) with rates (2) describe condensation in bosonic systems that are externally driven by a continuing supply of energy, dissipate into the environment and exhibit decoherence.

Equations of type (1) may also arise in atomic physics and quantum optics and are known as Pauli master equations^33^,^34^,^35^. They describe how the population of *S* non-degenerate energy levels changes over time when a system harbours *N* indistinguishable, non-interacting bosonic atoms. Such changes may occur by interactions with a radiation field that induces transitions between energy levels. A theoretical description of these transitions in terms of a Pauli master equation is appropriate if coherence is negligible. As in the previous example, the system then approaches a state in which some of the energy levels are macroscopically occupied (condensates), whereas the others are depleted. More generally, whenever a rate constant *r*_*ij*_ governs the transition of a single boson from a state *E*_*j*_ to *E*_*i*_, the rates (2) with *s*_*ij*_=1 for all *i* and *j* apply if *N* non-interacting bosons are brought into the system^19^.

### Strategy selection in evolutionary game theory

The classical master equation (1) also occurs in evolutionary game theory (EGT). Historically, EGT was developed to study the evolutionary processes that are driven by selection and mutation^36^,^37^ and seeks to identify optimal strategies for competitive interactions. For example, EGT has been applied in the study of the prominent ‘rock–paper–scissors' (RPS) game, which was proposed as a facilitator of species coexistence and has inspired both experimental and theoretical research^38^,^39^,^40^,^41^,^42^. Furthermore, the ‘prisoner's dilemma' game serves as a paradigmatic model to explore the evolution and maintenance of cooperation^43^,^44^. The interplay between non-linear and stochastic effects underlies the dynamics of such evolutionary games^45^,^46^,^47^,^48^,^49^,^50^.

In EGT, one typically considers a system of *N* interacting agents (classical particles) who repeatedly play one fixed strategy *E*_*i*_ out of the *S* possible choices 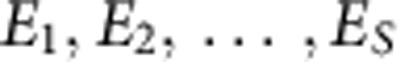. In each succeeding interaction, the defeated agent adopts the strategy of its opponent. Since *N*_*j*_ agents playing strategy *E*_*j*_ can potentially be defeated by one of the *N*_*i*_ agents playing strategy *E*_*i*_, the rate of change is Γ_*i*←*j*_=*r*_*ij*_*N*_*i*_*N*_*j*_. If an agent who plays *E*_*j*_ can also spontaneously mutate into an agent who plays *E*_*i*_ (with rate *μ*_*ij*_=*r*_*ij*_*s*_*ij*_), one recovers the classical master equation (1) with rates (2).

Thus, there exists a correspondence between condensation in incoherently driven-dissipative bosonic systems and strategy selection in EGT—the transition of bosons between states can be interpreted in terms of the interaction and mutation of agents employing evolutionary strategies. In effect, the states in an incoherently driven-dissipative set-up play an evolutionary game and the winning states form the condensates.

After having introduced the above examples, we now proceed with the mathematical and numerical analysis of the classical master equation (1). We show that the dynamics of condensation change on two distinct timescales. At the leading-order timescale, the dynamics are described by a set of non-linearly coupled, ordinary differential equations (see equation (3) below), which determine the states that become condensates. We identify these states by applying concepts from EGT. After an exposition of the physical principles that underlie the condensation dynamics, implications of our general results for incoherently driven-dissipative systems are discussed.

### The antisymmetric Lotka–Volterra equation

The total number of particles needed for condensation phenomena to occur is large (
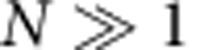
). To detect macroscopic occupancies, it is also assumed that the particle density *N*/*S* is large. Therefore, one may approximate the classical master equation (1) by a Langevin equation for the state concentrations *x*_*i*_(*t*)=*N*_*i*_(*t*)/*N* (details of the derivation are provided in Supplementary Note 1). Originally proposed for Brownian particles suspended in a liquid, the Langevin equation decomposes the dynamics of a sample trajectory of the random process into two contributions—into a deterministic drift and into noise stemming from the discreteness of particle numbers (‘demographic fluctuations'). Both the demographic fluctuations and the contribution to the deterministic drift that corresponds to mutations in the EGT setting are suppressed by a small prefactor 1/*N*. Therefore, these terms change the dynamics only slowly. The deterministic drift that corresponds to interactions between agents is, however, not suppressed. It thus governs the dynamics to leading order.

Hence, we find that the leading-order dynamics of the condensation process (1)–(2) are described by the differential equations:


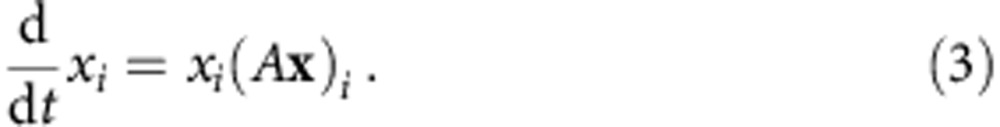


The matrix *A* is antisymmetric and encodes the effective transition rates between states (*a*_*ij*_=*r*_*ij*_–*r*_*ji*_). The constants *s*_*ij*_ that occur in the definition of the rates (2) do not change the leading-order dynamics, but they become relevant on subleading-order timescales.

We refer to equation (3) as the antisymmetric Lotka–Volterra equation (ALVE). It provides a description of pairwise interactions that preserve the total number of particles. Therefore, the ALVE finds a broad range of applications in diverse fields of research, in addition to the aforementioned condensation of bosons far from equilibrium. It was first studied by Volterra^51^ in the context of predator–prey oscillations in population biology^47^,^52^,^53^. In plasma physics, the ALVE describes the spectra of plasma oscillations (Langmuir waves)^54^,^55^, and in chemical kinetics it captures the dynamics of bimolecular autocatalytic reactions^18^,^56^,^57^,^58^. In EGT, the ALVE is known as the replicator equation of zero-sum games such as the RPS game^47^,^59^,^60^,^61^. Table 1 summarizes all of the above analogies.

Despite the simple structure of the ALVE, it exhibits a rich and complex behaviour. In the following, we show how the mathematical analysis of the ALVE explains condensation into multiple states (condensate selection). To this end, we extend an approach for the analysis of the ALVE that was introduced in the context of EGT^59^,^60^.

### Production of relative entropy and condensate selection

**Figure 1:**
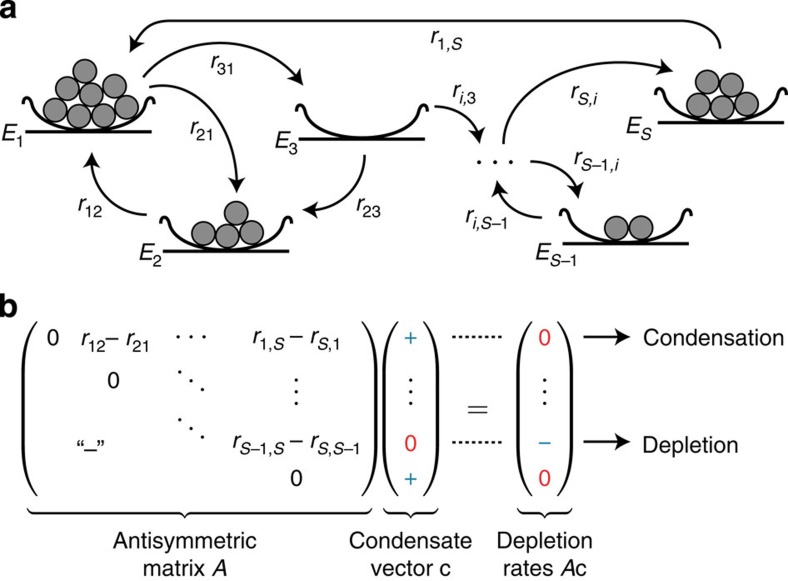
Condensation into multiple states due to particle transitions between states and mathematics of condensate selection. (**a**) With respect to condensation in an incoherently driven-dissipative quantum system, each bowl represents a state *E*_*i*_ that is occupied by *N*_*i*_ non-interacting bosons (filled circles). If indicated by an arrow, bosons may undergo transitions from state *E*_*j*_ to state *E*_*i*_ at a rate Γ_*i*←*j*_=*r*_*ij*_(*N*_*i*_+1)*N*_*j*_, with rate constant *r*_*ij*_. In the language of evolutionary game theory, the figure depicts the interaction of *N*_*i*_ agents (filled circles) playing strategies *E*_*i*_ (bowls) for *i*=1,...,*S*. An agent playing strategy *E*_*j*_ adopts strategy *E*_*i*_ at a rate Γ_*i*←*j*_=*r*_*ij*_*N*_*i*_*N*_*j*_. The above rate of bosonic condensate selection is recovered if agents may also spontaneously mutate from *E*_*j*_ to *E*_*i*_ at a rate *r*_*ij*_. (**b**) A condensate vector c for an antisymmetric matrix *A* has two properties: its entries are positive for indices for which *A*c is zero, and they are zero for indices for which *A*c is negative (“-'' signifies the antisymmetry of matrix *A*). Temporal evolution of the relative entropy of the condensate vector to the state concentrations under the ALVE (3) relates positive entries of the condensate vector to condensates, and its zero entries to depleted states. Generically, positive entries of c represent the asymptotic temporal average of oscillating condensate concentrations according to the ALVE (3), and negative entries of *A*c represent depletion rates.

Our analysis starts from a theorem in linear programming theory^62^. Given an antisymmetric matrix *A*, it is always possible to find a vector **c** that fulfils the following conditions: its entries are positive for indices in 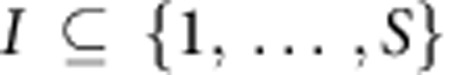 and zero for indices in 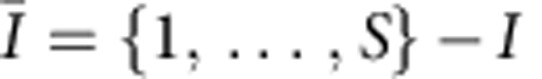, whereas the entries of *A***c** are zero for indices in *I* and negative for indices in 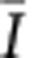 (Fig. 1b). Although several vectors **c** with these properties may exist, the index set *I* is unique and, thus, determined by the antisymmetric matrix *A*. Finding such a ‘condensate vector' **c** is crucial for the understanding of condensate selection and of the condensation dynamics. The condensate vector has the following physical interpretations.

All condensate vectors yield fixed points of the ALVE (3). Because of the antisymmetry of matrix *A*, a linear stability analysis of these fixed points does not yield insight into the global dynamics (Supplementary Note 1). However, the global stability properties can be inferred by showing that the relative entropy of a condensate vector to the state concentrations,


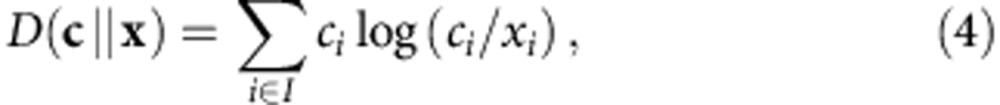


is a Lyapunov function (note that we do not consider the relative entropy of the state concentrations to the condensate vector, but define the relative entropy vice versa). The relative entropy (4) decreases with time and is bounded from below (see Methods and Supplementary Fig. 1). Therefore, the dynamics relax to a subsystem in which the relative entropy production is zero. The relaxation of relative entropy production is reminiscent of Prigogine's study of open systems in non-equilibrium thermodynamics. Indeed, we find that the system, to cite Prigogine's phrase, ‘settles down to the state of least dissipation'^63^.

**Figure 2:**
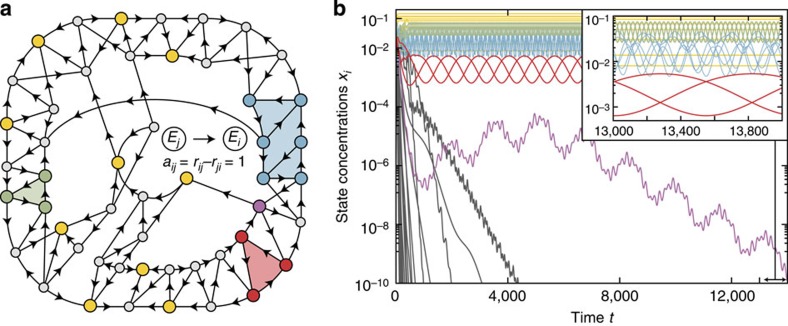
Fragmentation of an exemplary system into multiple condensates with oscillating state concentrations. (**a**) Randomly sampled network of 50 states. Disks represent states. An arrow from state *E*_*j*_ to state *E*_*i*_ represents an effective rate constant *a*_*ij*_=*r*_*ij*_–*r*_*ji*_=1 (a missing arrow indicates a forbidden transition with *a*_*ij*_=0). Computation of a condensate vector c predicted relaxation into 10 isolated condensates (yellow), one interacting subsystem with six condensates (blue) and two RPS cycles (red and green). All other states become depleted. The complete network also comprises RPS cycles of which some states become depleted. Knowledge of the network topology alone is thus insufficient to determine condensates. (**b**) Temporal evolution of state concentrations *x*_*i*_ (logarithmic scale). Colours in accordance with **a**. Numerical integration of the ALVE (3) confirmed the selection of states based on the condensate vector c. Subsystems with six (blue) and three (red and green) condensates exhibit oscillations of concentrations with non-vanishing particle flow. Depletion of states occurs exponentially fast. Identifying condensates from a condensate vector c is more reliable than through numerical integration: The concentration of the state associated to the purple disk in **a** decays exponentially to a concentration of 1.5 × 10^−7^ before recovering transiently. Numerical integration cannot rule out permanent recovery at later times. Supplementary Fig. 2 demonstrates such a case.

This state of least dissipation is characterized even further by the condensate vector **c**. Considering the definition of the relative entropy (4) and its boundedness, it follows that every concentration *x*_*i*_ with *i*∈*I* remains larger than a positive constant. On the other hand, states with indices in 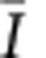 become depleted for long times (see Methods). Therefore, we find that the condensate vector determines the selection of condensates. Positive entries of **c** correspond to states that become condensates, whereas zero entries of **c** correspond to states that become depleted. Both the set of condensates and the set of depleted states are unique (Fig. 1b) and independent of the initial conditions. Generically, the entries of the condensate vector are also unique upon normalization (its entries sum up to one) and yield the rate |(*A***c**)_*i*_| at which a state *E*_*i*_ becomes depleted. The condensate selection occurs exponentially fast (see Fig. 2 and Methods).

After relaxation, the dynamics of the system are restricted to the condensates. In other words, the condensates form the attractor of the dynamics. However, the dynamics in this subsystem do not come to rest. The state of least dissipation is a dynamic state with a perpetually changing number of particles in the condensates—periodic, quasiperiodic and non-periodic oscillations are observed (Fig. 2b and Supplementary Fig. 1). In the generic case, the entries of the condensate vector represent the temporal average of condensate concentrations according to the ALVE (3). After condensate selection, the dynamics of these active condensates take place on a high-dimensional, deformed sphere^61^.

### An algebraic algorithm to find the condensates

Numerical integration of the ALVE (3) is neither a feasible nor a reliable method for identifying condensates (Fig. 2, Supplementary Figs 1 and 2). Instead, we determine these states by numerically searching for a condensate vector **c**. To this end, we reformulate the above conditions on **c** in terms of two linear inequalities^62^:


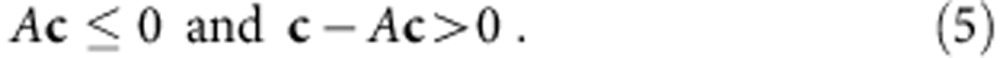


We solve these inequalities with a linear programming algorithm that is both reliable and efficient. The time to find a condensate vector scales only polynomially with the number of states *S* (see Supplementary Fig. 3 and Methods for details).

### Condensation in large random networks of states

We used our combined analytical and numerical approach to study how the connectivity of a random network of states affects the selection of condensates under the dynamics of the ALVE (3). The connectivity specifies the percentage of states between which particle transitions occur^20^,^64^. After having generated a network with a given connectivity, the strength and direction of an allowed transition between states *E*_*j*_ and *E*_*i*_ were determined by randomly sampling the corresponding effective rate constant *a*_*ij*_=*r*_*ij*_–*r*_*ji*_.

**Figure 3:**
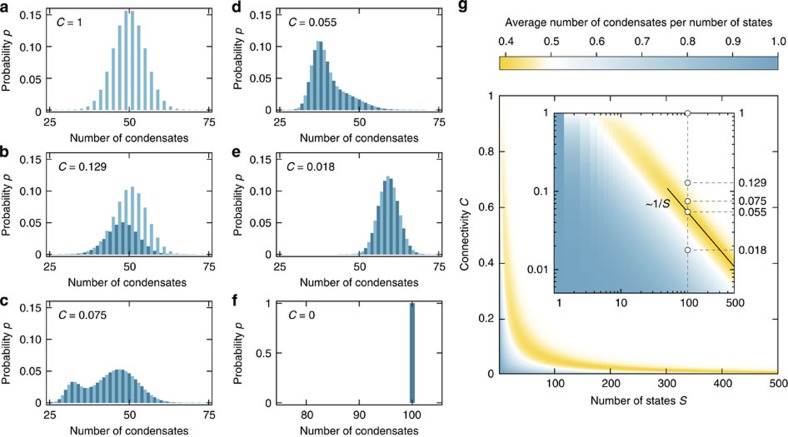
Dependence of the number of condensates on the connectivity of states in random networks. (**a**–**f**) Measured probability *p* of finding a particular number of condensates for a system with *S*=100 states and with connectivity *C* (5 × 10^6^ systems analysed per histogram). The connectivity specifies the percentage of states between which transitions of particles occur with a non-zero effective rate constant *a*_*ij*_=*r*_*ij*_–*r*_*ji*_. Effective rate constants *a*_*ij*_ were sampled from a Gaussian distribution (zero mean, unit variance). (**a**) At full connectivity, the distribution is pseudobinomial with only odd numbers of condensates (*C*=1; light blue bars)^19^,^60^,^66^. (**b**) As the connectivity is reduced, even numbers of condensates become possible when systems decouple into even numbers of subsystems (*C*=0.129; dark blue bars). (**c**) The distribution exhibits bimodality (*C*=0.075) and (**d**) approaches a minimal average number of 40.2 condensates (*C*=0.055). (**e**) This average subsequently increases (*C*=0.018) because isolated states are trivially selected as condensates (*C*=0) as shown in **f**. (**g**) Average number of condensates per number of states (colour coded) plotted against the number of states *S* and the connectivity *C* (log–log graph in inset; ≥10^4^ systems per data point, see Supplementary Fig. 4 for the reliability of the linear programming algorithm). White circles correspond to distributions shown in (**a**–**e**). The minimal relative number of condensates conforms to the power law *C*∼1/*S*^*γ*^ with *γ*=0.998±0.008 (s.e.m.) and can be related to the criticality of random networks^20^.

Our results for condensation in large random networks of states are summarized in Fig. 3. When the connectivity of a network is zero, all of its states are isolated. Particles are not exchanged between states and none of the states becomes depleted. For an increased connectivity, isolated pairs of states are sampled in a random network. One state in an isolated pair is always depleted and the average number of condensates decreases rapidly. On approaching a critical connectivity, cycles and trees of all orders become embedded in a random network. This critical connectivity scales inversely with the number of states^20^. We observe that, under the dynamics of the ALVE (3), the average number of condensates becomes minimal for a connectivity that also scales inversely with the number of states (see Fig. 3g). We attribute this minimum to the interplay between the criticality of random networks and condensate selection on connected components of the network^61^,^65^. Embedded directed cycles are a recurring motif^21^ in the remaining network of condensates after condensate selection. Above the critical connectivity, a single giant cluster is formed. On average, half the number of states in this giant cluster become condensates once the network is fully connected (*C*=1; Fig. 3a)^19^,^60^,^66^. Thus, our analysis emphasizes the importance of critical properties of random networks for condensate selection.

### Design of active condensates

**Figure 4:**
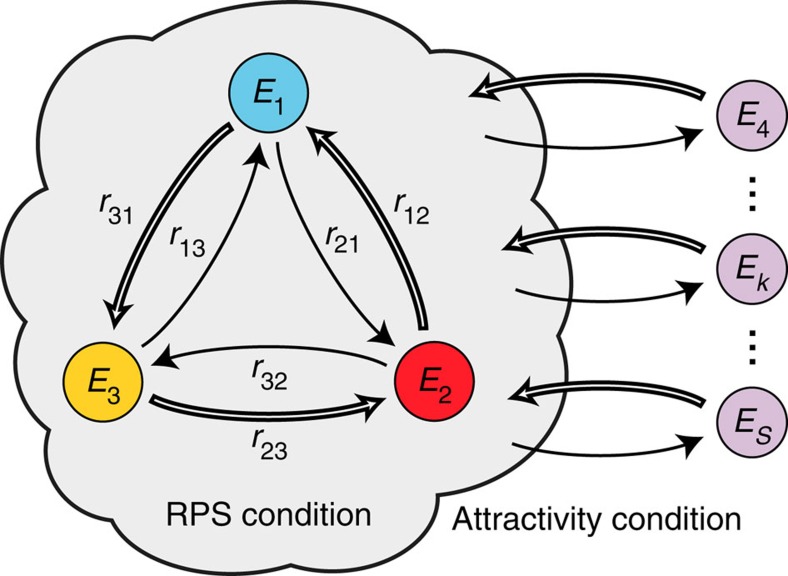
Conditions for the emergence of a RPS cycle of condensates. Three particular states *E*_1_, *E*_2_ and *E*_3_ (blue, red and yellow disks) of a network condense into a RPS cycle if, and only if, two conditions are fulfilled: First, the ‘RPS condition' requires that the rate constants *r*_*ij*_ between the three states form a RPS network: *r*_*i*−1,*i*+1_>*r*_*i*+1,*i*−1_ (indices are counted modulo 3, for example, *r*_42_=*r*_12_ (framed arrows denote rate constants that are larger than rate constants for the respective reverse direction). Differences between these rate constants define the entries *c*_*i*_=*r*_*i*−1,*i*+1_–*r*_*i*+1,*i*−1_ of an admissible condensate vector c. Second, the ‘attractivity condition' requires that the weighted sum of rates from any exterior state *E*_*k*_ (purple disks) into the RPS cycle, 
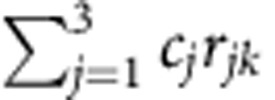
 (framed arrows), is larger than the weighted sum of outbound rates, 
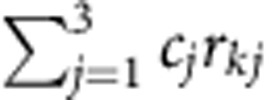
 (black arrows). In other words, the inflow of particles into the RPS cycle from any exterior state needs to be greater than the outflow to that state.

Our understanding of the condensate selection can be used to design systems that condense into a particular network of states, a game of condensates. We exemplify this procedure by formulating conditions under which a system relaxes into a RPS game of condensates^40^. Three particular states *E*_1_, *E*_2_ and *E*_3_ in a system become a RPS cycle of condensates if, and only if, the following two conditions are fulfilled (Fig. 4). First, the ‘RPS condition' requires that the rate constants between the three states form a RPS network (for example, *r*_12_>*r*_21_, *r*_23_>*r*_32_ and *r*_31_>*r*_13_). Second, the ‘attractivity condition' requires that the inflow of particles into the RPS cycle from any other state *E*_*k*_ is greater than the outflow to that state *E*_*k*_ (for all 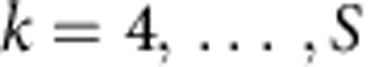). The values of the rate constants between the states that become depleted are irrelevant. More complex games of condensates can be designed by formulating similar conditions on the rate constants. These conditions are formulated as inequalities that depend on Pfaffians of the antisymmetric matrix *A* and its submatrices (see Methods)^61^. The flow of particles between states in these systems causes condensate concentrations to oscillate (Fig. 2b).

## Discussion

Our findings thus suggest intriguing dynamics of condensates in systems whose temporal evolutions are captured by the classical master equation (1) with rates (2), for example, in driven-dissipative systems of non-interacting bosons. Condensates observed on the leading-order timescale are metastable. For longer times, relaxation into a steady state occurs^19^,^67^. When detailed balance is broken in the system of condensates, the net probability current between at least two states does not vanish and a non-equilibrium steady state is approached^68^,^69^. The simplest way of designing such condensates is illustrated by the above RPS game. In this game, detailed balance is broken, for example, when the transition of particles is unidirectional (with totally asymmetric rate constants *r*_12_>*r*_21_=0, *r*_23_>*r*_32_=0, and *r*_31_>*r*_13_=0). For non-interacting bosons in driven-dissipative systems, the continuing supply with energy through the external time-periodic driving force (Floquet system) and the dissipation of energy into the environment may, therefore, prevent the system from reaching equilibrium. How such systems may be realized in an experiment poses an interesting question for future research.

The transition of particles between condensates in the here-studied coupled birth–death processes parallels the interaction and mutation of winning agents in evolutionary game theory, reflecting an ‘evolutionary game of condensates'. Our results suggest the possibility of creating novel bosonic systems with an oscillating occupation of condensates. Non-interacting bosons in incoherently driven-dissipative systems are promising candidates. Since the antisymmetric Lotka-Volterra equation also arises in population biology, chemical kinetics and plasma physics, all of our mathematical results apply to these fields as well.

## Methods

### Asymptotics of the ALVE

The asymptotic behaviour of the ALVE (3) can be characterized as follows: for every antisymmetric matrix *A* there exists a unique subset of states 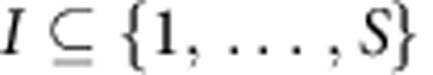 whose concentrations stay away from zero for all times, that is,


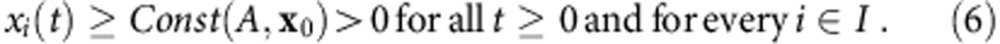


The set *I* is the set of condensates. All of the other states with indices in 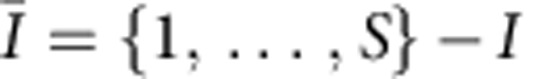 become depleted as *t*→∞, that is,


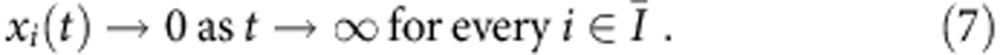


The set of condensates can be determined algebraically from the antisymmetric matrix *A* and does not depend on the initial conditions 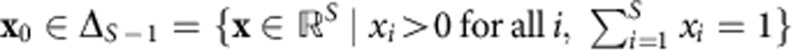.

To show this result, the time-dependent entropy *D*(**c**||**x**)(*t*) of a condensate vector 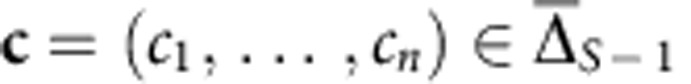 (*c*_*i*_≥0 for all *i* and 
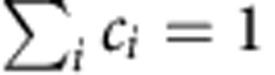
) relative to the trajectory **x**(*t*) is considered (that is, the Kullback–Leibler divergence of **x**(*t*) from **c**), see equation (4). A condensate vector is defined via the properties (see Fig. 1b):


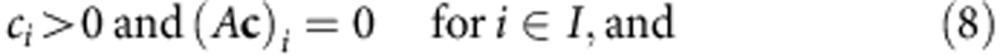



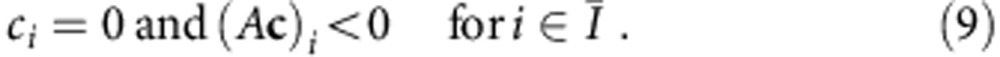


Such a vector can always be found for an antisymmetric matrix^62^. Notably, the index set *I* is unique although more than one condensate vector may exist.

Considering the time derivative of the relative entropy *D*(**c**||**x**)(*t*) and employing equations (3) and (8) yields:


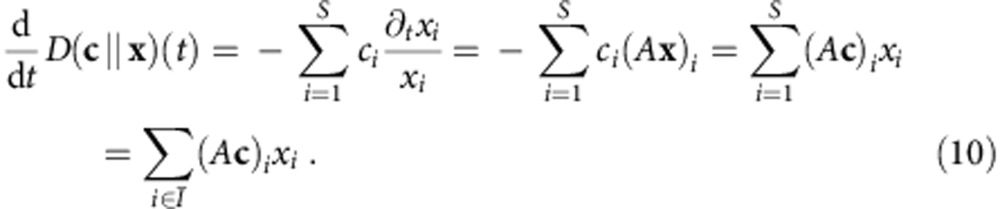


Since 
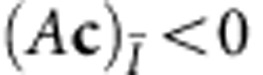
 and **x**>0, it follows that *∂*_*t*_*D*(**c**||**x**)(*t*)<0 (please note the overbars in subscripts, which may be lost when read in low resolution). Therefore, the relative entropy *D*(**c**||**x**) is a Lyapunov function if **c** is chosen in accordance with equations (8) and (9). Moreover, *D*(**c**||**x**) is bounded from above by *D*(**c**||**x**)(0) and from below by zero for all times. This can be seen from the definition of *D*, and from the integration of equation (10) (using that 
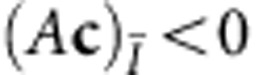
 and **x**>0):


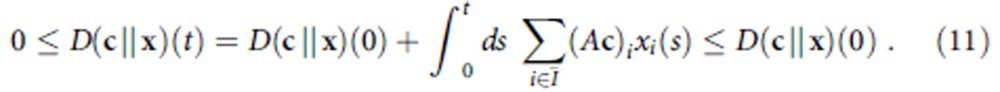


From the definition of the relative entropy in equation (4), it follows that every concentration *x*_*i*_ with *i*∈*I* remains larger than a positive constant, that is, *x*_*i*_(*t*)≥*Const*(*A*, **x**_0_)>0 for all times *t* (if *x*_*i*_(*t*)→0 for *i*∈*I*, it follows that *D*→∞, which contradicts the boundedness of *D*).

Furthermore, equation (11) implies that,


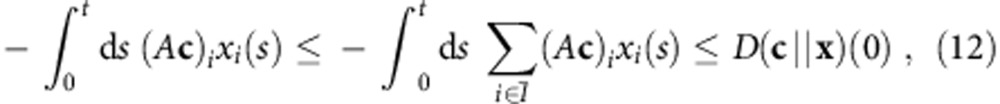


for every 
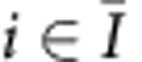
 and for all *t*. Therefore, concentration *x*_*i*_ is integrable for every 
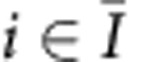
 (*x*_*i*_∈*L*^1^(0,∞)) with the bound:


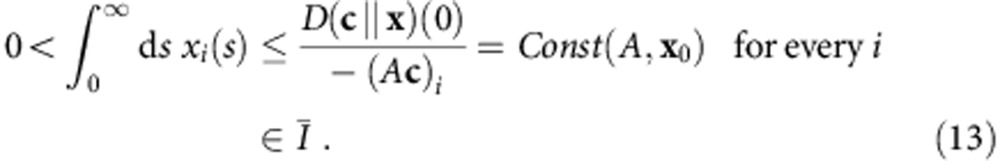


Since the derivative of the concentrations is bounded from above and below, |*∂*_*t*_*x*_*i*_|=|*x*_*i*_(*A***x**)_*i*_|≤ || (*A***x**) ||_∞_≤ || *A* ||_∞→∞_≤Const(*A*), one concludes that *x*_*i*_ is uniformly continuous (|| *A* ||_∞→∞_ denotes the operator norm of *A* induced by the maximum norm on 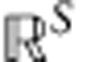). Together with the integrability (13), it follows that the states with indices in 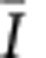 become depleted as *t*→∞, that is, *x*_*i*_(*t*)→0 for 
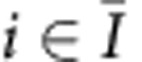
.

In conclusion, given an antisymmetric matrix *A*=*R*–*R*^*T*^ via a rate constant matrix *R*={*r*_*ij*_}_*i,j*_, one finds a condensate vector **c** that satisfies inequalities (8)–(9). The index set *I*, for which the entries of **c** are positive, represents condensates. The index set 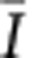, for which entries of **c** are zero, represents states that become depleted. Moreover, equation (10) implies that the relative entropy becomes a conserved quantity in the subsystem of condensates (Supplementary Fig. 1).

### Temporal average of condensate concentrations

The ALVE (3) is solved implicitly by,


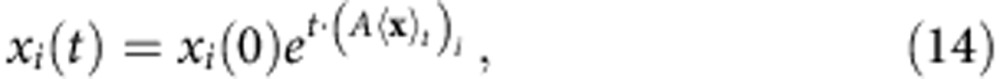


with the time average of the trajectory 〈**x**〉_*t*_ defined as:


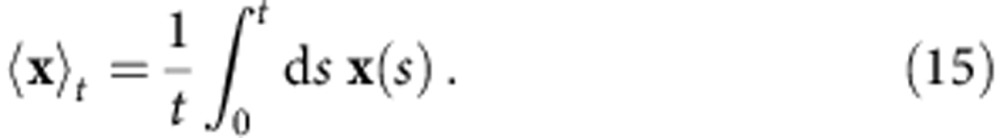


It is shown above that 0<*Const*(*A*, **x**_0_)≤*x*_*i*_(*t*)≤1 holds for the states that become condensates (*i*∈*I*). By rearranging equation (14), one thus obtains:


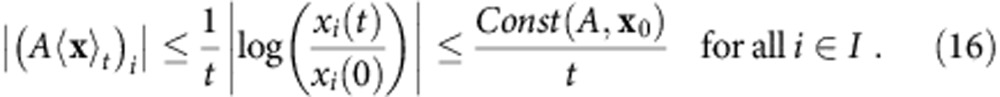


Note that *Const* is used to denote arbitrary positive, time-independent constants. Therefore, the right-hand side of equation (16) vanishes for *t*→∞. On the other hand, *x*_*i*_ is integrable for 
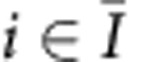
 (equation (13)). Thus, the corresponding component of the time average converges to zero,


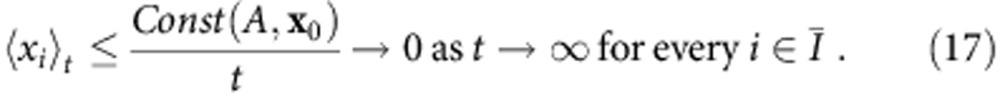


Hence, the distance of the time average 〈**x**〉_*t*_ to the kernel of the antisymmetric submatrix *A*^*I*^ converges to zero (the submatrix *A*^*I*^ corresponds to the system of condensates with indices in *I*).

### Structure of a generic antisymmetric matrix

For systems with an even number of states *S*, the antisymmetric matrix *A*=*R*–*R*^*T*^ generically has a trivial kernel, whereas for systems with an odd number of states, the kernel of *A* is generically one dimensional. A higher dimensional kernel of *A* only occurs if the matrix entries are tuned^52^,^61^,^70^. As a consequence, when all of the entries above the diagonal of *A* are, for example, randomly drawn from a continuous probability distribution (for example from a Gaussian distribution), all 2^*S*^ submatrices of *A* have a kernel with dimension of less than or equal to one.

The projection of 
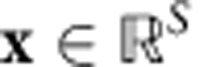
 to the subspace 
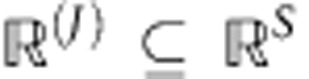
 for an arbitrary index set 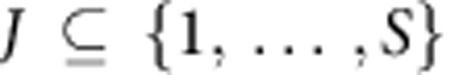 is defined as **x**_*J*_:=*P*_*J*_**x**:=(*x*_*j*_)_*j*∈*J*_. In other words, entries of **x**_*J*_ are zero for indices in the complement 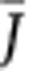. In the following, the short notation *A*^*J*^:=*P*_*J*_*AP*_*J*_ is also used (see above). Furthermore, the set of antisymmetric matrices whose submatrices have a kernel with dimension ≤1 is defined:


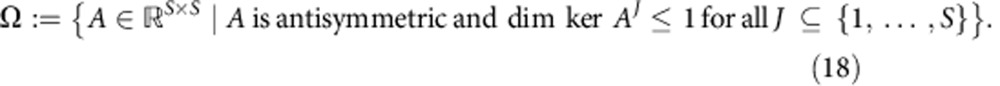


The complement 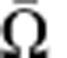 has measure zero with respect to the flat measure d*A* on antisymmetric matrices (the translation invariant measure, which is sigma-finite and not trivial).

For antisymmetric matrices *A*∈Ω, the kernel can be characterized as follows^61^,^70^. If the number of states *S* is even, the kernel of *A* is trivial: ker *A*={**0**}. If the number of states is odd, the kernel is one-dimensional: ker *A*={**v**}. This kernel element can be computed analytically in terms of Pfaffians of submatrices of *A*:


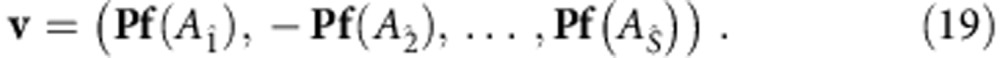


The submatrix 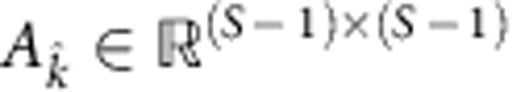 denotes the matrix for which the *k*-th column and row are removed from *A*.

For antisymmetric matrices *A*∈Ω, the normalized condensate vector **c** with 
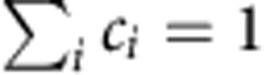
 and with properties (8), (9) is unique. The latter follows from *A*^*I*^**c**=0 (equations (8) and (18)). Therefore, the condensate vector is the unique kernel vector of the subsystem of condensates whose interactions are characterized by the matrix *A*^*I*^. Furthermore, *I* contains an odd number of elements. To determine the condensate vector for *A*∈Ω, one can proceed as follows. For each odd-dimensional submatrix *A*^*I*^ with 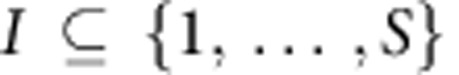, one computes the kernel element **v** according to equation (19) and defines the vector 
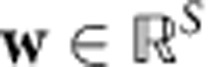
 by setting **w**_*I*_=**v** and 
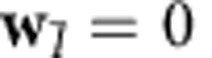
. There exists exactly one set *I* for which 
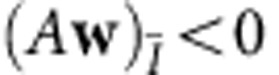
. The corresponding vector **w** is the unique condensate vector upon normalization.

### Temporal average of condensate concentrations (generic case)

It was shown above that the temporal average of condensate concentrations 〈**x**〉_*t*_ converges to a non-negative kernel element of the antisymmetric matrix *A*^*I*^. In the generic case, the condensate vector **c** is the unique kernel element of *A*^*I*^ upon normalization. Therefore, positive entries of **c** represent the asymptotic temporal average of condensate concentrations,


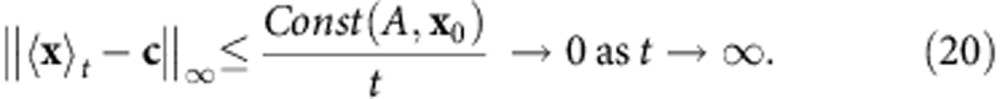


### Exponentially fast depletion of states (generic case)

On inserting equation (20) into the implicit solution (14) of the ALVE, the exponentially fast depletion of states with 
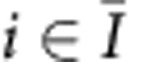
 can be seen as follows (note that (*A***c**)_*i*_<0 according to the choice of the condensate vector in equations (8) and (9)):


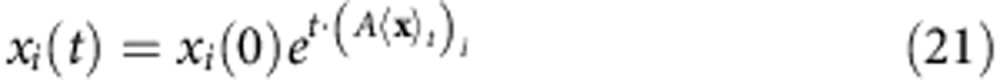



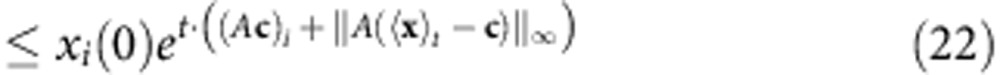



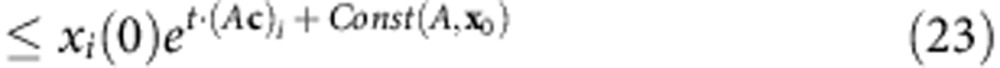



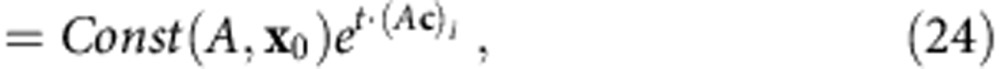


and analogously,


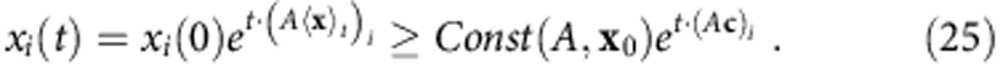


Therefore, condensate selection occurs exponentially fast at depletion rate |(*A***c**)_*i*_|. The dynamics of cases for non-generic antisymmetric matrices are discussed in Supplementary Note 2.

### Linear programming algorithm

For the numeric computation of condensate vectors **c**, a finite threshold *δ*>0 was introduced into the inequalities (5): *A***c**≤0 and **c**–*A***c**≥*δ*>0. Its value was set to *δ*=1 by rescaling of **c**. Numerical solution of the inequalities was performed by using the IBM ILOG CPLEX Optimization Studio 12.5 and its interface to the C++ language. The software Mathematica 9.0 from Wolfram Research was also found to be applicable. Further information on the calibration of the linear programming algorithm and a simplified Mathematica algorithm are provided in Supplementary Note 3.

## Author contributions

J.K., M.F.W., T.K. and E.F. designed, discussed and planned the study. T.K., J.K. and M.F.W. developed the analytical results. M.F.W., T.K. and J.K. developed the numerical algorithms and generated the data. J.K., M.F.W., T.K. and E.F. interpreted the results and wrote the manuscript.

## Additional information

**How to cite this article:** Knebel, J. *et al.* Evolutionary games of condensates in coupled birth–death processes. *Nat. Commun.* 6:6977 doi: 10.1038/ncomms7977 (2015).

## Supplementary Material

Supplementary Information Supplementary Figures 1-4, Supplementary Notes 1-3 and Supplementary References

## Figures and Tables

**Table 1:** Condensation processes described by the ALVE in different fields of research.

**Field of research**	**Entity**	**State**	**Process**	**Dynamics**
Quantum physics^19^	Boson	Quantum state	Incoherent transition	Condensation/depletion
Evolutionary game theory^59^,^60^	Agent	Strategy	Game (+mutation)	Win/loss
Population dynamics^47^,^51^,^52^,^53^	Individual	Species	Competition (+mutation)	Survival/extinction
Chemical kinetics^56^,^57^,^58^	Molecule	Chemical species	Reaction (+conversion)	Production/consumption
Plasma physics^54^,^55^	Plasmon	Jet	Scattering	Increase/decrease

ALVE, antisymmetric Lotka–Volterra equation.

The ALVE (3) governs condensation processes in diverse fields of research. For example, for incoherently driven-dissipative bosonic systems, the ALVE describes condensation and depletion of states by incoherent transitions of non-interacting bosons. In EGT, the ALVE occurs in the context of winning and losing strategies played by agents.

## References

[b1] Krapivsky P. L., Redner S. & Leyvraz F. Connectivity of growing random networks. Phys. Rev. Lett. 85, 4629–4632 (2000). 11082613 10.1103/PhysRevLett.85.4629

[b2] Bianconi G. & Barabási A.-L. Bose-Einstein condensation in complex networks. Phys. Rev. Lett. 86, 5632–5635 (2001). 11415319 10.1103/PhysRevLett.86.5632

[b3] Evans M. R. & Hanney T. Nonequilibrium statistical mechanics of the zero-range process and related models. J. Phys. A: Math. Gen. 38, R195–R240 (2005).

[b4] Evans M. R. Bose-Einstein condensation in disordered exclusion models and relation to traffic flow. Europhys. Lett. 36, 13–18 (1996).

[b5] Krug J. & Ferrari P. A. Phase transitions in driven diffusive systems with random rates. J. Phys. A: Math. Gen. 29, L465–L471 (1996).

[b6] Chowdhury D., Santen L. & Schadschneider A. Statistical physics of vehicular traffic and some related systems. Phys. Rep. 329, 199–329 (2000).

[b7] Kaupužs J., Mahnke R. & Harris R. J. Zero-range model of traffic flow. Phys. Rev. E 72, 056125 (2005). 10.1103/PhysRevE.72.056125 16383706

[b8] Spitzer F. Interaction of Markov processes. Adv. Math. 5, 246–290 (1970).

[b9] Evans M. R. & Waclaw B. Condensation in stochastic mass transport models: beyond the zero-range process. J. Phys. A: Math. Theor. 47, 095001 (2014).

[b10] Bose S. N. Plancks Gesetz und Lichtquantenhypothese. Z. Phys. 26, 178–181 (1924).

[b11] Einstein A. Quantentheorie des einatomigen idealen Gases. Sitzb. d. Preuss. Akad. d. Wiss 261–267 (1924).

[b12] Einstein A. Quantentheorie des einatomigen idealen Gases. Zweite Abhandlung. Sitzb. d. Preuss. Akad. d. Wiss 3–14 (1925).

[b13] Griffin A., Snoke D. & Stringari G. Bose Einstein Condensation Cambridge Univ. Press (1995).

[b14] Anglin J. R. & Ketterle W. Bose-Einstein condensation of atomic gases. Nature 416, 211–218 (2002). 11894104 10.1038/416211a

[b15] Penrose O. & Onsager L. Bose-Einstein condensation and liquid helium. Phys. Rev. 104, 576–584 (1956).

[b16] Mueller E. J., Ho T.-L., Ueda M. & Baym G. Fragmentation of Bose-Einstein condensates. Phys. Rev. A 74, 033612 (2006).

[b17] Gardiner C. Stochastic Methods: A Handbook for the Natural and Social Sciences Springer (2009).

[b18] Van Kampen N. G. Stochastic Processes in Physics and Chemistry Elsevier (2007).

[b19] Vorberg D., Wustmann W., Ketzmerick R. & Eckardt A. Generalized Bose-Einstein condensation into multiple states in driven-dissipative systems. Phys. Rev. Lett. 111, 240405 (2013). 24483633 10.1103/PhysRevLett.111.240405

[b20] Albert R. & Barabási A.-L. Statistical mechanics of complex networks. Rev. Mod. Phys. 74, 47–97 (2002).

[b21] Milo R. *et al.* Network motifs: simple building blocks of complex networks. Science 298, 824–827 (2002). 12399590 10.1126/science.298.5594.824

[b22] Blümel R. *et al.* Dynamical localization in the microwave interaction of Rydberg atoms: the influence of noise. Phys. Rev. A 44, 4521–4540 (1991). 9906493 10.1103/physreva.44.4521

[b23] Kohler S., Dittrich T. & Hänggi P. Floquet-Markovian description of the parametrically driven, dissipative harmonic quantum oscillator. Phys. Rev. E 55, 300–313 (1997).

[b24] Breuer H.-P., Huber W. & Petruccione F. Quasistationary distributions of dissipative nonlinear quantum oscillators in strong periodic driving fields. Phys. Rev. E 61, 4883–4889 (2000). 10.1103/physreve.61.4883 11031530

[b25] Grifoni M. & Hänggi P. Driven quantum tunneling. Phys. Rep. 304, 229–354 (1998).

[b26] Breuer H.-P. & Petruccione F. The Theory of Open Quantum Systems Oxford Univ. Press (2002).

[b27] Gardiner C. W. & Zoller P. Quantum kinetic theory: a quantum kinetic master equation for condensation of a weakly interacting Bose gas without a trapping potential. Phys. Rev. A 55, 2902–2921 (1997).

[b28] Kagan Y. & Svistunov B. V. Evolution of correlation properties and appearance of broken symmetry in the process of Bose-Einstein condensation. Phys. Rev. Lett. 79, 3331–3334 (1997).

[b29] Bijlsma M. J., Zaremba E. & Stoof H. T. C. Condensate growth in trapped Bose gases. Phys. Rev. A 62, 063609 (2000).

[b30] Gardiner C. W., Lee M. D., Ballagh R. J., Davis M. J. & Zoller P. Quantum kinetic theory of condensate growth: comparison of experiment and theory. Phys. Rev. Lett. 81, 5266–5269 (1998).

[b31] Walser R., Williams J., Cooper J. & Holland M. Quantum kinetic theory for a condensed bosonic gas. Phys. Rev. A 59, 3878–3889 (1999).

[b32] Kocharovsky V. V., Scully M. O., Zhu S.-Y. & Suhail Zubairy M. Condensation of N bosons. II. Nonequilibrium analysis of an ideal Bose gas and the laser phase-transition analogy. Phys. Rev. A 61, 023609 (2000).

[b33] Pauli W. Festschrift zum 60. Geburtstage A. Sommerfeld Hirzel (1928).

[b34] Mandel L. & Wolf E. Optical Coherence and Quantum Optics Cambridge Univ. Press (1995).

[b35] Gardiner C. W. & Zoller P. Quantum Noise Springer (2004).

[b36] Maynard Smith J. Evolution and the Theory of Games Cambridge Univ. Press (1982).

[b37] Nowak M. A. & Sigmund K. Evolutionary dynamics of biological games. Science 303, 793–799 (2004). 14764867 10.1126/science.1093411

[b38] Sinervo B. & Lively C. M. The rock-paper-scissors game and the evolution of alternative male strategies. Nature 380, 240–243 (1996).

[b39] Kerr B., Riley M., Feldman M. & Bohannan B. Local dispersal promotes biodiversity in a real-life game of rock-paper-scissors. Nature 418, 171–174 (2002). 12110887 10.1038/nature00823

[b40] Reichenbach T., Mobilia M. & Frey E. Mobility promotes and jeopardizes biodiversity in rock-paper-scissors games. Nature 448, 1046–1049 (2007). 17728757 10.1038/nature06095

[b41] Weber M. F., Poxleitner G., Hebisch E., Frey E. & Opitz M. Chemical warfare and survival strategies in bacterial range expansions. J. R. Soc. Interface 11, 20140172 (2014). 24806706 10.1098/rsif.2014.0172 PMC4032534

[b42] Szolnoki A. *et al.* Cyclic dominance in evolutionary games: a review. J. R. Soc. Interface 11, 20140735 (2014). 25232048 10.1098/rsif.2014.0735 PMC4191105

[b43] Nowak M. A., Sasaki A., Taylor C. & Fudenberg D. Emergence of cooperation and evolutionary stability in finite populations. Nature 428, 646–650 (2004). 15071593 10.1038/nature02414

[b44] Szolnoki A., Antonioni A., Tomassini M. & Perc M. Binary birth-death dynamics and the expansion of cooperation by means of self-organized growth. EPL 105, 48001 (2014).

[b45] McKane A. J. & Newman T. J. Predator-prey cycles from resonant amplification of demographic stochasticity. Phys. Rev. Lett. 94, 218102 (2005). 16090353 10.1103/PhysRevLett.94.218102

[b46] Traulsen A., Claussen J. C. & Hauert C. Coevolutionary dynamics: from finite to infinite populations. Phys. Rev. Lett. 95, 238701 (2005). 16384353 10.1103/PhysRevLett.95.238701

[b47] Reichenbach T., Mobilia M. & Frey E. Coexistence versus extinction in the stochastic cyclic Lotka-Volterra model. Phys. Rev. E 74, 51907 (2006). 10.1103/PhysRevE.74.051907 17279939

[b48] Melbinger A., Cremer J. & Frey E. Evolutionary game theory in growing populations. Phys. Rev. Lett. 105, 178101 (2010). 21231082 10.1103/PhysRevLett.105.178101

[b49] Biancalani T., Dyson L. & McKane A. J. Noise-induced bistable states and their mean switching time in foraging colonies. Phys. Rev. Lett. 112, 038101 (2014). 24484166 10.1103/PhysRevLett.112.038101

[b50] Rulands S., Jahn D. & Frey E. Specialization and bet hedging in heterogeneous populations. Phys. Rev. Lett. 113, 108102 (2014). 25238387 10.1103/PhysRevLett.113.108102

[b51] Volterra V. Leçons sur la Théorie Mathématique de la Lutte pour la Vie Gauthier-Villars (1931).

[b52] Goel N. S., Maitra S. C. & Montroll E. W. On the Volterra and other nonlinear models of interacting populations. Rev. Mod. Phys. 43, 231–276 (1971).

[b53] May R. M. Stability and Complexity in Model Ecosystems Princeton Univ. Press (1973). 4723571

[b54] Zakharov V., Musher S. & Rubenchik A. Nonlinear stage of parametric wave excitation in a plasma. JETP Lett. 19, 151–152 (1974).

[b55] Manakov S. Complete integrability and stochastization of discrete dynamical systems. Sov. Phys.-JETP 40, 269–274 (1975).

[b56] Itoh Y. Boltzmann equation on some algebraic structure concerning struggle for existence. Proc. Jpn Acad. 47, 854–858 (1971).

[b57] Di Cera E., Phillipson P. E. & Wyman J. Chemical oscillations in closed macromolecular systems. Proc. Natl Acad. Sci. USA 85, 5923–5926 (1988). 3413066 10.1073/pnas.85.16.5923 PMC281877

[b58] Di Cera E., Phillipson P. E. & Wyman J. Limit-cycle oscillations and chaos in reaction networks subject to conservation of mass. Proc. Natl Acad. Sci. USA 86, 142–146 (1989). 2911564 10.1073/pnas.86.1.142 PMC286420

[b59] Akin E. & Losert V. Evolutionary dynamics of zero-sum games. J. Math. Biol. 20, 231–258 (1984). 6542123 10.1007/BF00275987

[b60] Chawanya T. & Tokita K. Large-dimensional replicator equations with antisymmetric random interactions. J. Phys. Soc. Jpn 71, 429–431 (2002).

[b61] Knebel J., Krüger T., Weber M. F. & Frey E. Coexistence and survival in conservative Lotka-Volterra networks. Phys. Rev. Lett. 110, 168106 (2013). 23679644 10.1103/PhysRevLett.110.168106

[b62] Kuhn H. & Tucker A. Linear Inequalities and Related Systems Princeton Univ. Press (1956).

[b63] Prigogine I. Time, structure, and fluctuations. Science 201, 777–785 (1978). 17738519 10.1126/science.201.4358.777

[b64] Gardner M. R. & Ashby W. R. Connectance of large dynamic (cybernetic) systems: critical values for stability. Nature 228, 784 (1970). 5472974 10.1038/228784a0

[b65] Durney C. H., Case S. O., Pleimling M. & Zia R. K. P. Saddles, arrows, and spirals: deterministic trajectories in cyclic competition of four species. Phys. Rev. E 83, 051108 (2011). 10.1103/PhysRevE.83.051108 21728491

[b66] Allesina S. & Levine J. M. A competitive network theory of species diversity. Proc. Natl Acad. Sci. USA 108, 5638–5642 (2011). 21415368 10.1073/pnas.1014428108 PMC3078357

[b67] Eisert J., Friesdorf M. & Gogolin C. Quantum many-body systems out of equilibrium. Nat. Phys. 11, 124–130 (2015).

[b68] Zia R. K. P. & Schmittmann B. Probability currents as principal characteristics in the statistical mechanics of non-equilibrium steady states. J. Stat. Mech 2007, P07012 (2007).

[b69] Kriecherbauer T. & Krug J. A pedestrian's view on interacting particle systems, KPZ universality and random matrices. J. Phys. A: Math. Theor. 43, 403001 (2010).

[b70] Cullis C. E. Matrices and Determinoids vol. I and II, Cambridge Univ. Press (1913).

