# Supplementary material for: Evolutionary games of condensates in coupled birth–death processes
Source: Nat Commun. 2015 Apr 24;6:6977. doi: 10.1038/ncomms7977 (PMC4421816; doi:10.1038/ncomms7977)
Supplement: Supplementary Information — Supplementary Figures 1-4, Supplementary Notes 1-3 and Supplementary References [file ncomms7977-s1.pdf]

SUPPLEMENTARY FIGURE 1

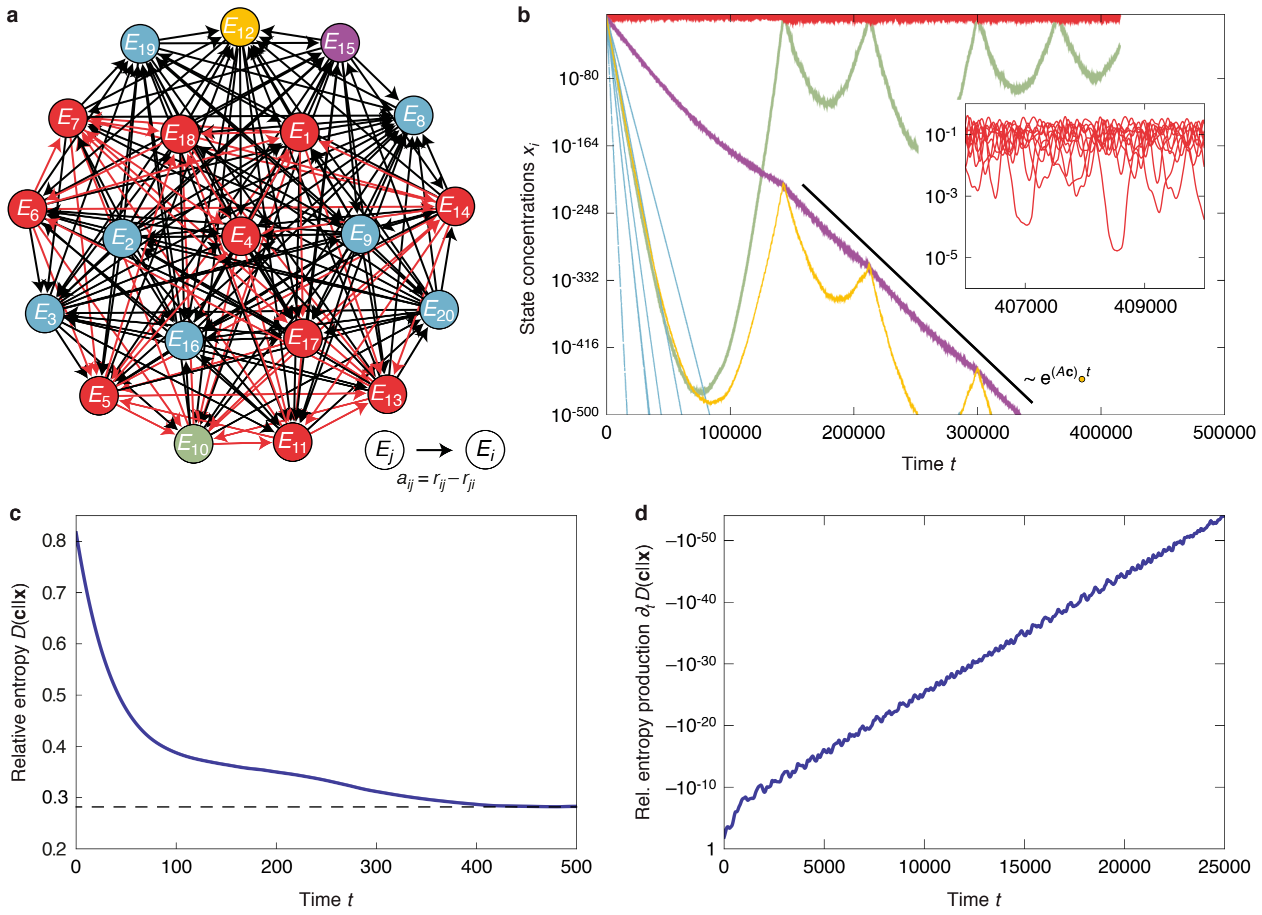

Supplementary Figure 1. Advantage of the algebraic algorithm over numerical integration of the antisymmetric Lotka-Volterra equation (ALVE). (**a**) A fully connected network with 20 states (colored disks). Effective transition rates a_{ij} (arrows) are listed in Supplementary Note 3 and were sampled from a Gaussian distribution (zero mean, unit variance). Computation of the unique normalized condensate vector **c** identifies 11 states as condensates (red and green disks) and depletion of 9 states (blue, yellow, and purple disks). (**b**) Trajectories show the temporal evolution of state concentrations x_i (colors in accordance with (**a**)). Numerical integration of the ALVE is highly unstable. Only the routine NDSolve with method "StiffnessSwitching" of Mathematica from Wolfram Research was able to track the concentrations for a sufficiently long time. Routines offered by the GNU Scientific Library failed (Dormand-Prince and Runge-Kutta-Fehlberg). The concentration corresponding to the green state transiently decreases to a value of $4 \cdot 10^{-473}$ before recovering. Its asymptotic temporal average is given by the corresponding entry of the condensate vector **c** as $1.6 \cdot 10^{-4}$. This average could, however, not be verified by integration due to numerical failure at $t = 414960$. Negative entries of A**c** determine the rates of exponentially fast depletion as illustrated by the black line for the yellow state (note the logarithmic scaling). (**c**) Temporal evolution of the relative entropy $D(\mathbf{c}||\mathbf{x})(t)$ (blue line). The relative entropy decreases towards a non-zero asymptotic value. (**d**) The production of relative entropy $\partial_t D(\mathbf{c}||\mathbf{x})(t)$ (blue line) is, therefore, negative and vanishes for large times.

SUPPLEMENTARY FIGURE 2

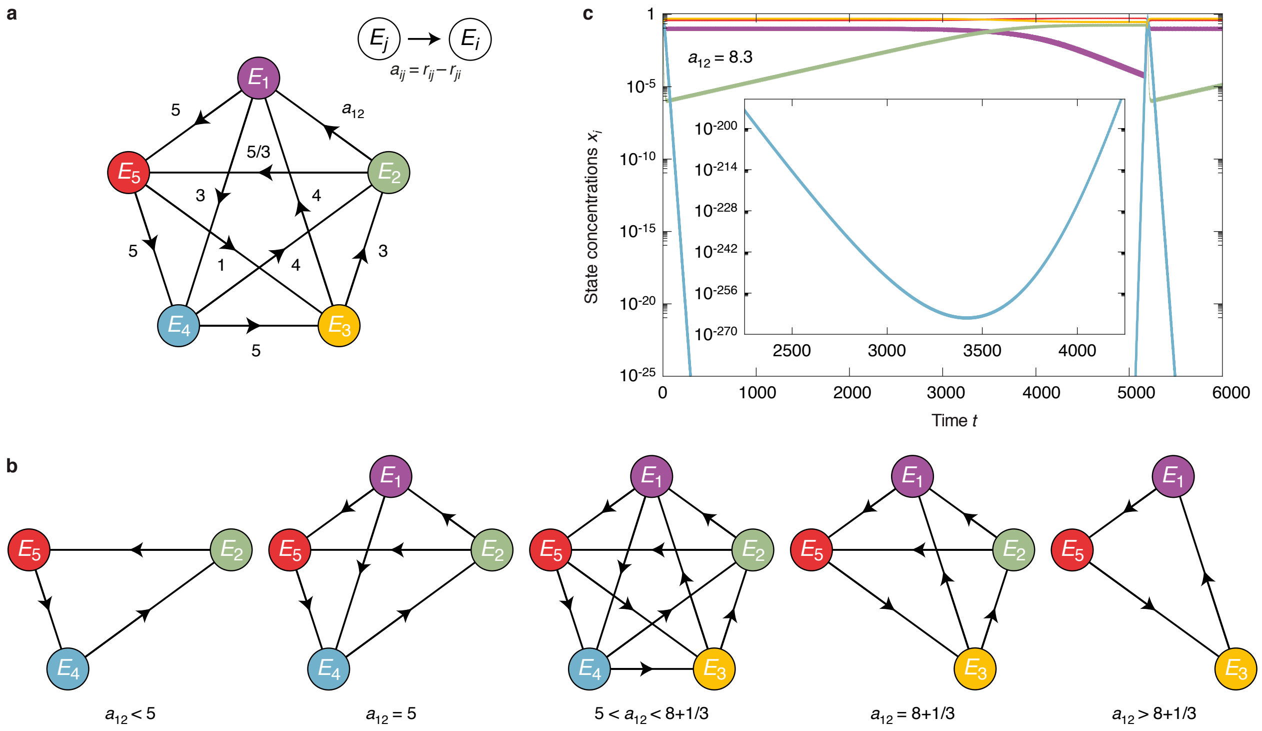

Supplementary Figure 2. Identification of condensates for a system with five states. (**a**) Colored disks represent states E_i. An arrow from E_j to E_i denotes an effective rate constant $a_{ij} = r_{ij} - r_{ji}$. (**b**) Computation of condensate vectors for different values of a_{12} yields depletion of states E_1 and E_3 for $a_{12} < 5$, depletion of state E_3 for $a_{12} = 5$, condensation of all states for $5 < a_{12} < 8 + 1/3$, depletion of state E_4 for $a_{12} = 8 + 1/3$, and depletion of states E_2 and E_4 for $a_{12} > 8 + 1/3$. These results can be verified by using the Mathematica code supplied in Supplementary Note 3. (**c**) Numerical integration of the ALVE confirms the selection of condensates, but becomes error-prone in the vicinity of values of a_{12} at which the set of condensates changes. The trajectories were obtained for $a_{12} = 8.3$, which is slightly smaller than the value at which E_4 becomes depleted. Identification of condensates from trajectories requires the introduction of a threshold for concentrations below which states are considered as depleted. If such a threshold is set to values larger than $\sim 2.5 \cdot 10^{-265}$, state E_4 is erroneously considered to be depleted, despite its periodic recovery.

SUPPLEMENTARY FIGURE 3

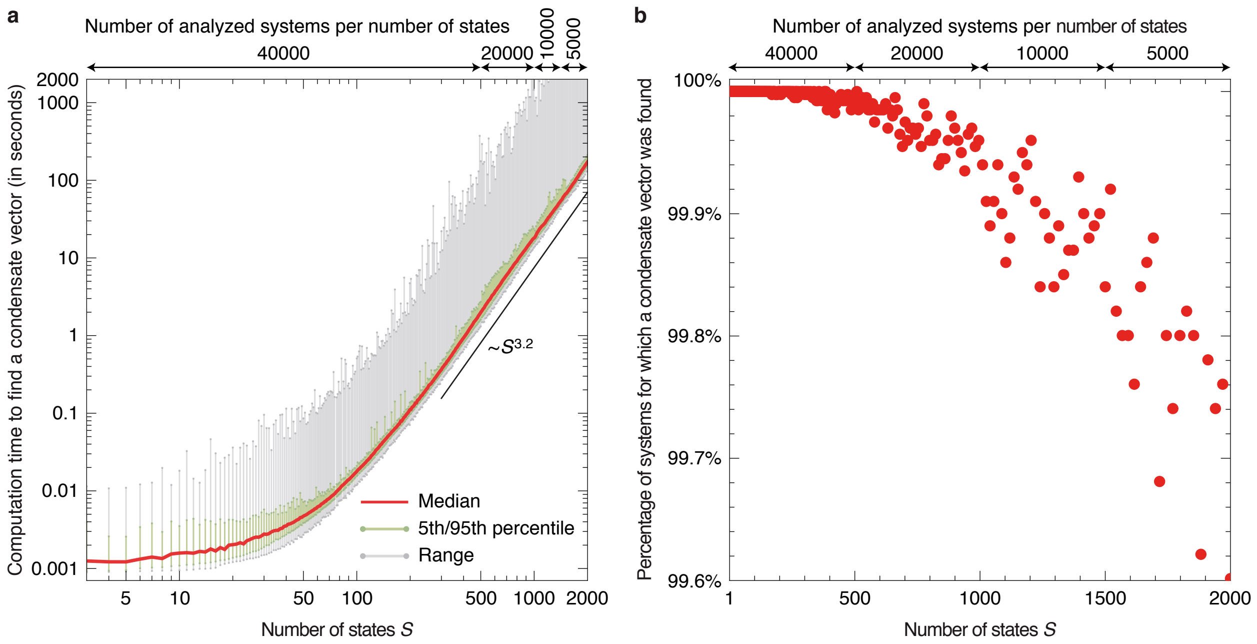

Supplementary Figure 3. Run-time analysis of the linear programming algorithm for fully connected random networks of states. The computation time to find a condensate vector was measured for systems with up to $S = 2000$ states. The systems were constructed by sampling effective rate constants $a_{ij} = r_{ij} - r_{ji}$ from a Gaussian distribution (zero mean, unit variance). The numbers of systems (ensemble size) that were analyzed for different numbers of states S are indicated at the top. (**a**) The median of computation times is shown in red, the regime between the 5th and 95th percentiles in green, and the range of computation times in grey (log-log graph). Computation time increases at most polynomially as indicated by the black line (linear fit of the median with exponent 3.221 ± 0.008 (s.e.m.)). (**b**) Percentage of networks for which a condensate vector was found (red dots). The lowest percentage was 99.6%. All computations were performed on machines with 10 Intel Xeon E5-2670v2 cores (2.50 GHz) and 128 GB RAM. Parallelization was not used in CPLEX.

SUPPLEMENTARY FIGURE 4

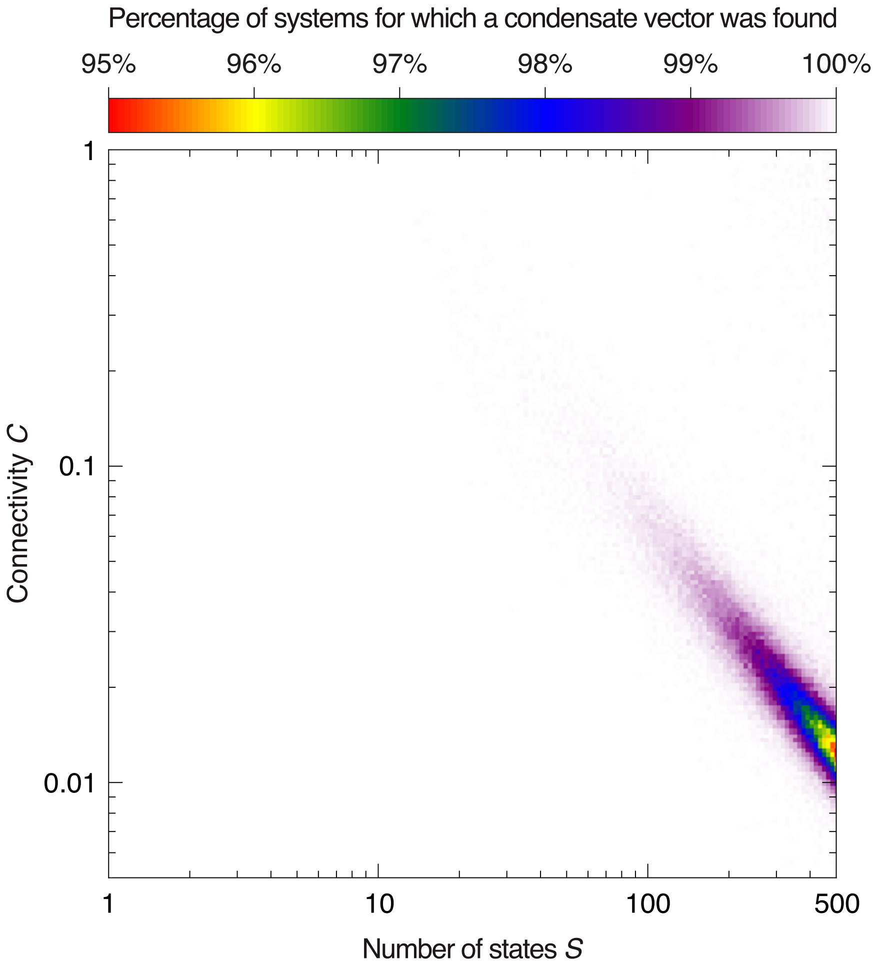

Supplementary Figure 4. Reliability of the linear programming algorithm for large random networks. Color-coded representation of the percentage of random networks with S states and connectivity C for which the linear programming algorithm found a suitable condensate vector ($\geq 10^4$ analyzed systems per data point; the figure accompanies Fig. 3b). The percentage was close to 100% for most parameters but decreased in the vicinity of the power law shown in Fig. 3b (lowest percentage: 94.9% for networks with $S = 500$ states and connectivity $C = 0.013$).

SUPPLEMENTARY NOTE 1: APPROXIMATION OF THE CLASSICAL MASTER EQUATION BY STOCHASTIC DIFFERENTIAL EQUATIONS AND OCCURRENCE OF THE ANTISYMMETRIC LOTKA-VOLTERRA EQUATION

In the following, we supplement the discussion of the classical master equation that governs condensation of non-interacting bosons in an incoherently driven-dissipative system. When the total number of particles in the system is large ($N \gg 1$), the classical master equation can be approximated by a Fokker-Planck equation. This Fokker-Planck equation is rewritten as an Itō stochastic differential equation, which is equivalent to a Langevin equation. From the analysis of the stochastic differential equation, we find that the leading order dynamics of the condensation process is governed by the antisymmetric Lotka-Volterra equation (equation (3) in the main text).

Classical master equation for the coupled birth-death processes with conservation of total particle number

We consider a system of S non-degenerate states $E_i, i = 1, \dots, S$, each of which is occupied by N_i indistinguishable particles (Fig. 1a). The configuration of the system at time t is fully characterized by the vector of occupation numbers $\mathbf{N} = (N_1, N_2, \dots, N_S) \in \mathbb{Z}^S$ with $N_i \geq 0$. It changes only due to coupled creation (birth) and annihilation (death) processes between two connected states E_i and E_j:

$$(N_1, \dots, N_i - 1, \dots, N_j + 1, \dots, N_S) \leftrightarrows (N_1, \dots, N_i, \dots, N_j, \dots, N_S) \leftrightarrows (N_1, \dots, N_i + 1, \dots, N_j - 1, \dots, N_S)\,. \tag{1}$$

We introduce the following short-hand notation for these processes:

$$\mathbf{N} - \mathbf{e}_i + \mathbf{e}_j \quad \leftrightarrows \quad \mathbf{N} \quad \leftrightarrows \quad \mathbf{N} + \mathbf{e}_i - \mathbf{e}_j\,. \tag{2}$$

Here, the vector $\mathbf{e}_i \in \mathbb{Z}^S$ denotes the unit vector in direction i (equal to one at index i, otherwise zero). The above creation and annihilation processes conserve the total number of particles $N = \sum_i N_i$. We are interested in the probability $P(\mathbf{N}, t)$ of finding the system in configuration $\mathbf{N}$ at time t, given that it was initially in configuration $\mathbf{N}_0$ at time t_0. The temporal evolution of the probability distribution $P(\mathbf{N}, t)$ is governed by the classical master equation (equation (1) in the main text):

$$\partial_t P(\mathbf{N}, t) = \sum_{\substack{i,j=1 \\ j \neq i}}^{S} \Big(\Gamma_{i \leftarrow j}(N_i - 1, N_j + 1) P(\mathbf{N} - \mathbf{e}_i + \mathbf{e}_j, t) - \Gamma_{i \leftarrow j}(N_i, N_j) P(\mathbf{N}, t) \Big)\,. \tag{3}$$

In our work, we consider the following transition rate from configuration $\mathbf{N}$ to configuration $\mathbf{N} + \mathbf{e}_i - \mathbf{e}_j$:

$$\Gamma_{i \leftarrow j}(N_i, N_j) = r_{ij}(N_i + s_{ij}) N_j, \text{ with } s_{ij} \geq 0 \text{ and } r_{ij} \geq 0\,. \tag{4}$$

This transition rate encompasses the two model classes of condensate selection in bosonic systems and in evolutionary game theory (EGT) as described in the main text. In the context of bosonic condensation, the parameters s_{ij} are equal to 1 for all i and j, whereas it may assume any non-negative value in the context of EGT. In EGT, s_{ij} contributes to the mutation or switching rate [1].

For the study of incoherently driven-dissipative systems of non-interacting bosons, the above description in terms of a classical master equation requires that the system under consideration is weakly coupled to a reservoir [2–5]. The reservoir has to be such that correlations in it decay rapidly. In particular, the Born-Markov and the rotating wave approximation are assumed in the derivation of the classical master equation. These assumptions imply that off-diagonal entries in the reduced density operator of the system decay fast enough such that coherence in the quantum system is negligible. In addition, the initial states of the system and the reservoir should not be correlated after their preparation.

These conditions are, for example, fulfilled in systems of non-interacting bosons that are both periodically driven in time (Floquet systems) and weakly coupled to a thermal bath (see Vorberg et al. [2] for a detailed discussion).

Derivation of the Fokker-Planck equation

In the following, we approximate the classical master equation (3) in the limit of a large number of particles ($N \gg 1$) [6]. For that purpose, we introduce state concentrations $\mathbf{x} = (x_1, \ldots, x_S)$ with $x_i = N_i/N$. The concentrations are intensive variables and elements of the $(S-1)$-dimensional open simplex $\Delta_{S-1} = \{\mathbf{x} \in \mathbb{R}^S \mid x_i > 0 \text{ for all } i,\ \sum_{i=1}^S x_i = 1\}$. In the limit $N \to \infty$, they become continuous variables. We denote their corresponding probability distribution by $p(\mathbf{x}, t)$. Furthermore, we rescale time by $t \to t/N$. It can be straightforwardly seen that the following formulation of the classical master equation for $p(\mathbf{x}, t)$ is equivalent to the form given in equation (3):

$$\partial_t p(\mathbf{x}, t) = \sum_{\substack{i,j=1 \\ j \neq i}}^{S} \int_{\mathbb{R}} d\Delta x_i \int_{\mathbb{R}} d\Delta x_j \sum_{k \in \{0,1\}} N^{1-k} \Big(\gamma^{(k)}(\Delta x_i, \Delta x_j; x_i - \Delta x_i, x_j - \Delta x_j)\, p(\mathbf{x} - \Delta x_i \mathbf{e}_i - \Delta x_j \mathbf{e}_j, t) - \gamma^{(k)}(\Delta x_i, \Delta x_j; x_i, x_j)\, p(\mathbf{x}, t) \Big) , \tag{5}$$

$$\text{with} \quad \gamma^{(0)}(\Delta x_i, \Delta x_j; x_i, x_j) = r_{ij} x_i x_j\, \delta(\Delta x_i - 1/N) \delta(\Delta x_j + 1/N) , \tag{6}$$

$$\text{and} \quad \gamma^{(1)}(\Delta x_i, \Delta x_j; x_i, x_j) = r_{ij}\, s_{ij}\, x_j\, \delta(\Delta x_i - 1/N) \delta(\Delta x_j + 1/N) . \tag{7}$$

This classical master equation can be approximated by performing a Kramers-Moyal expansion. Truncation of the expansion at second order, leads to the Fokker-Planck equation:

$$\partial_t p(\mathbf{x}, t) = -\sum_{i=1}^{S} \partial_i \big(\alpha_i(\mathbf{x}) p(\mathbf{x}, t) \big) + \frac{1}{2N} \sum_{i,j=1}^{S} \partial_i \partial_j \big(\beta_{ij}(\mathbf{x}) p(\mathbf{x}, t) \big) . \tag{8}$$

Here, $\alpha(\mathbf{x})$ is the drift vector and $\beta(\mathbf{x})$ the diffusion matrix.

The drift vector is given by:

$$\alpha_i(\mathbf{x}) = \sum_{\substack{j=1 \\ j \neq i}}^{S} \int_{\mathbb{R}} d\Delta x_i \int_{\mathbb{R}} d\Delta x_j \sum_{k \in \{0,1\}} \frac{1}{N^k} \Big(\gamma^{(k)}(\Delta x_i, \Delta x_j; x_i, x_j) + \gamma^{(k)}(\Delta x_j, \Delta x_i; x_j, x_i) \Big) N \Delta x_i , \tag{9}$$

$$= x_i \sum_{j=1}^{S} (r_{ij} - r_{ji}) x_j + \frac{1}{N} \sum_{j=1}^{S} (r_{ij} s_{ij} x_j - r_{ji} s_{ji} x_i) =: \alpha_{i,0}(\mathbf{x}) + \frac{1}{N} \alpha_{i,1}(\mathbf{x}) . \tag{10}$$

At leading order in $1/N$ (that is, at order $\mathcal{O}(1)$), the drift vector is determined by the antisymmetric part $A = R - R^T$ of the rate constant matrix $R = \{r_{ij}\}_{i,j}$. In other words, the matrix A is antisymmetric with entries $a_{ij} = r_{ij} - r_{ji}$.

For the diffusion matrix, we have to distinguish between its diagonal entries,

$$\beta_{ii}(\mathbf{x}) = \sum_{\substack{j=1\\ j\neq i}}^{S} \int_{\mathbb{R}} d\Delta x_i \int_{\mathbb{R}} d\Delta x_j \sum_{k\in\{0,1\}} \frac{1}{N^k}\Big(\gamma^{(k)}(\Delta x_i, \Delta x_j; x_i, x_j) + \gamma^{(k)}(\Delta x_j, \Delta x_i; x_j, x_i)\Big) N^2 \Delta x_i^2 \; , \tag{11}$$

$$= \sum_{\substack{j=1\\ j\neq i}}^{S} (r_{ij} + r_{ji}) x_i x_j + \frac{1}{N} \sum_{\substack{j=1\\ j\neq i}}^{S} (r_{ij} s_{ij} x_j + r_{ji} s_{ji} x_i) =: \beta_{ii,0}(\mathbf{x}) + \frac{1}{N}\beta_{ii,1}(\mathbf{x}) \; , \tag{12}$$

and its off-diagonal entries ($i \neq j$):

$$\beta_{ij}(\mathbf{x}) = \int_{\mathbb{R}} d\Delta x_i \int_{\mathbb{R}} d\Delta x_j \sum_{k\in\{0,1\}} \frac{1}{N^k}\Big(\gamma^{(k)}(\Delta x_i, \Delta x_j; x_i, x_j) + \gamma^{(k)}(\Delta x_j, \Delta x_i; x_j, x_i)\Big) N^2 \Delta x_i \Delta x_j \; , \tag{13}$$

$$= -(r_{ij} + r_{ji}) x_i x_j - \frac{1}{N}\big(r_{ij} s_{ij} x_j + r_{ji} s_{ji} x_i\big) =: \beta_{ij,0}(\mathbf{x}) + \frac{1}{N}\beta_{ij,1}(\mathbf{x}) \; . \tag{14}$$

Both diagonal and off-diagonal entries of the diffusion matrix are determined by the symmetric part of the rate constant matrix R at leading order in $1/N$.

Derivation of the stochastic differential equations

The Fokker-Planck equation (8) can be transformed into a system of Itō stochastic differential equations (SDEs) [6]:

$$\mathrm{d}x_i = \alpha_i(\mathbf{x})\, \mathrm{d}t + \frac{1}{\sqrt{N}} \sum_{j=1}^{S} \zeta_{ij}(\mathbf{x})\, \mathrm{d}W_j \; . \tag{15}$$

Here, $\mathrm{d}W_j$ represents a Wiener increment of zero mean and unit variance. The matrix $\zeta(\mathbf{x})$ is a square root of the diffusion matrix $\beta(\mathbf{x})$ in the sense that $\zeta\zeta^T = \beta$ (the diffusion matrix β is positive semi-definite). Although ζ is not unique, its choice does not change the stochastic nature of the process (an orthogonal transformation $\zeta \to \zeta\mathcal{T}$ with $\mathcal{T}\mathcal{T}^T = \mathbb{I}_S$ does not change the corresponding Fokker-Planck equation). The decomposition $\beta_{ij} = \beta_{ij,0} + \frac{1}{N}\beta_{ij,1}$ in equations (11) and (13) implies that ζ can be written as $\zeta_{ij} = \zeta_{ij,0} + \mathcal{O}\big(\frac{1}{N}\big)$.

The Itō SDEs (15) can also be written in Langevin form, which are often used in the physics literature [6, 7]:

$$\frac{\mathrm{d}}{\mathrm{d}t} x_i = \alpha_i(\mathbf{x}) \; + \frac{1}{\sqrt{N}} \sum_{j=1}^{S} \zeta_{ij}(\mathbf{x})\, \eta_j \; . \tag{16}$$

Here, η_j represents uncorrelated Gaussian white noise.

The antisymmetric Lotka-Volterra equation

We identify a leading (fast) and a subleading (slow) timescale of the SDE (15). On the leading timescale ($t \sim \mathcal{O}(1)$), only the drift term $\alpha_{i,0}$ is relevant, whereas on the subleading timescale ($t \sim \mathcal{O}(N)$), the terms $\alpha_{i,1}$ and $\zeta_{ij,0}$ compete. The latter terms cause only slow changes on the leading $\mathcal{O}(1)$-timescale. In other words, the dynamics on the subleading timescale cause only slow changes of the $\mathcal{O}(1)$-trajectory.

More specifically, we find that at order $t \sim \mathcal{O}(1)$, only $\alpha_{i,0}$ determines the change in concentrations such that:

$$\frac{\mathrm{d}}{\mathrm{d}t} x_i = x_i \sum_j (r_{ij} - r_{ji}) x_j = x_i (A\mathbf{x})_i \ . \tag{17}$$

As stated in the main text, we refer to this equation as the antisymmetric Lotka-Volterra equation (ALVE). The initial concentrations are assumed to lie in the open simplex Δ_{S-1}, that is $\mathbf{x}(t=0) =: \mathbf{x}_0 \in \Delta_{S-1}$. We note that the dynamics defined by equation (17) cannot leave the simplex, that is $\mathbf{x}(t) \in \Delta_{S-1}$ for all times [8].

We note that the van Kampen system size expansion [9] of the master equation (3) yields the same deterministic equation (17) as our derivation via Fokker-Planck and Langevin equation at the leading order timescale.

Stability in the linear approximation around fixed points of the ALVE

We discuss the fixed points $\mathbf{x}^* \in \overline{\Delta}_{S-1}$ ($x_i^* \geq 0$ and $\sum_i x_1^* = 1$) of the ALVE (17), that is the points for which the dynamics is stationary ($\partial_t x_i^* = x_i^* (A\mathbf{x}^*)_i = 0$). In the following, we show that a linear stability analysis of these fixed points does not yield insight into the global dynamics of the ALVE.

First, every condensate vector $\mathbf{c}$ (normalized such that $\sum_i c_i = 1$) of the antisymmetric matrix A yields a fixed point of the ALVE. This can be seen from the properties of a condensate vector $\mathbf{c}$ (see Methods section of the main text) [10]:

$$c_i > 0 \ \text{ and } (A\mathbf{c})_i = 0 \text{ for } i \in I\text{, and} \tag{18}$$

$$c_i = 0 \ \text{ and } (A\mathbf{c})_i < 0 \text{ for } i \in \bar{I} \ . \tag{19}$$

Notably, the index set I is unique although more than one condensate vector may exist. Furthermore, there exist fixed points $\mathbf{x}^* \in \overline{\Delta}_{S-1}$ and a different index set $J \neq I$ for which $x_j^* > 0$ and $(A\mathbf{x}^*)_j = 0$ for $j \in J$, and $x_j^* = 0$ for $j \in \bar{J}$ but $(A\mathbf{x}^*)_j < 0$ does not hold for all $j \in \bar{J}$ (in other words, condition (18) is fulfilled, but condition (19) is not).

We first study the stability in the linear approximation around the fixed points that are given by condensate vectors. Upon introducing the distance $\Delta\mathbf{x}$ of a normalized condensate vector $\mathbf{x}^* = \mathbf{c}$ from the concentrations $\mathbf{x}$ as a new variable, $\Delta\mathbf{x} := \mathbf{x} - \mathbf{c}$, one obtains from the ALVE (17) the temporal behavior of that translated variable as follows:

$$\frac{\mathrm{d}}{\mathrm{d}t}\Delta x_i = \Delta x_i (A\mathbf{c})_i + c_i (A\Delta\mathbf{x})_i + \Delta x_i (A\Delta\mathbf{x})_i \ \text{, that is,} \tag{20}$$

$$\text{for } i \in I: \ \frac{\mathrm{d}}{\mathrm{d}t}\Delta x_i = \sum_{j=1}^{S} c_i a_{ij} \Delta x_j + R(\Delta\mathbf{x}) \ , \tag{21}$$

$$\text{for } i \in \bar{I}: \ \frac{\mathrm{d}}{\mathrm{d}t}\Delta x_i = (A\mathbf{c})_i \Delta x_i + R(\Delta\mathbf{x}) \ , \tag{22}$$

with $R(\Delta\mathbf{x}) = \Delta x_i (A\Delta\mathbf{x})_i = \mathcal{O}(\|\Delta\mathbf{x}\|^2)$.

Next, we discuss the stability of the condensate vectors in the linear approximation (linear stability analysis of fixed points). The cardinality of the set I is referred to as $|I|$. After relabeling of the indices, one obtains up to linear order in $\|\Delta\mathbf{x}\|$:

$$\frac{\mathrm{d}}{\mathrm{d}t}\begin{pmatrix}\Delta\mathbf{x}_I \\ \Delta\mathbf{x}_{\bar{I}}\end{pmatrix} = \begin{pmatrix}\tilde{A}_c & B \\ 0 & \tilde{A}_s\end{pmatrix}\begin{pmatrix}\Delta\mathbf{x}_I \\ \Delta\mathbf{x}_{\bar{I}}\end{pmatrix} =: \tilde{A}\Delta\mathbf{x}\,, \tag{23}$$

with $\tilde{A}_c$ denoting the $(|I| \times |I|)$-dimensional matrix with elements $(\tilde{A}_c)_{i,j} = c_i a_{ij}$ for $i, j \in I$. $\tilde{A}_s$ denotes the diagonal, $((S - |I|) \times (S - |I|))$-dimensional matrix with entries $(\tilde{A}_s)_{i,j} = (A\mathbf{c})_i \delta_{ij}$ for $i, j \in \bar{I}$. The matrix B is of dimension $|I| \times (S - |I|)$ with elements $B_{ij} = c_i a_{ij}$ for $i \in I$ and $j \in \bar{I}$.
The eigenvalues of the matrix $\tilde{A}$ determine the linear stability of the fixed points. Because of the block upper triangular structure of the matrix $\tilde{A}$, its eigenvalues are given by the eigenvalues of the matrices $\tilde{A}_s$ and $\tilde{A}_c$. All eigenvalues of the diagonal matrix $\tilde{A}_s$ are negative because $(A\mathbf{c})_i < 0$ for $i \in \bar{I}$. All eigenvalues of the matrix $\tilde{A}_c$ have vanishing real part. The latter can be seen from defining the nonsingular, $(|I| \times |I|)$-dimensional matrix V with elements $(V)_{i,j} = c_i \delta_{ij}$ for $i, j \in I$. Since the matrix A^I is an antisymmetric matrix, all eigenvalues of $\tilde{A}_c = VA^I$ are purely imaginary as well (the $(|I| \times |I|)$-dimensional submatrix A^I corresponds to the system of condensates with indices in I; see Methods section of the main text). However, the matrix $\tilde{A}_c$ is not antisymmetric in general. This argument can be seen as follows (see for example the Appendix in [11]): Consider the diagonal, nonsingular matrix $V^{1/2}$, whose square is the matrix V, and whose inverse is the matrix $V^{-1/2}$. The matrix $V^{-1/2}(VA^I)V^{1/2}$ has the same eigenvalues as the matrix VA^I. Since the matrix $V^{-1/2}(VA^I)V^{1/2} = V^{1/2}A^I V^{1/2}$ is antisymmetric and, thus, has purely imaginary eigenvalues, also the matrix VA^I has purely imaginary eigenvalues.

Consequently, the fixed points of the ALVE (17) that are given by the condensate vectors possess a $(S - |I|)$-dimensional local, invariant stable manifold $\mathcal{M}_s$ and a $|I|$-dimensional local, invariant center manifold $\mathcal{M}_c$ (see for example Theorem 3.2.1 in [12]). Concentrations with initial conditions chosen in $\mathcal{M}_s$ decay to zero exponentially fast. However, for initial concentrations that do not lie in $\mathcal{M}_s$, the temporal behavior cannot be inferred ("linearly stable solutions may be nonlinearly unstable" [12]).

We note that the above linear stability analysis applies to any fixed point of the ALVE. Any fixed point of the ALVE possesses an at least $|J|$-dimensional local, invariant center manifold $\mathcal{M}_c$ (see definition of J above). Therefore, a linear stability analysis of the fixed points of the ALVE (17) does not yield insight into the global dynamics of the ALVE, at least not in the straightforward fashion.

SUPPLEMENTARY NOTE 2: DYNAMICS OF CASES FOR NON-GENERIC ANTISYMMETRIC MATRICES

Some of the results presented in the main text for generic antisymmetric matrices ($A \in \Omega$) can be extended to matrices with higher dimensional kernels. Here, Ω is defined as in equation (18) of the main text as the set of antisymmetric matrices whose submatrices have a kernel with dimension less than one or equal to one:

$$\Omega = \left\{ A \in \mathbb{R}^{S \times S} \mid A \text{ is antisymmetric and } \dim \ker A^J \leq 1 \text{ for all } J \subseteq \{1, \ldots, S\} \right\} . \tag{24}$$

When submatrices of A have a kernel of dimension greater than or equal to two ($A \notin \Omega$) the statements generalize as follows: The temporal average of the projection to the surviving concentrations converges to the positive kernel of the attractive subsystem. This convergence takes place on a time scale which is not slower than $1/t$. More precisely, the distance between the time average of the concentrations of the selected states and the kernel of the surviving subsystem tends to zero for large times:

$$\text{dist}\left(\langle \mathbf{x}_I \rangle_t , \ker A^I \right) \leq \frac{Const(A, \mathbf{x}_0)}{t} . \tag{25}$$

Typically, one still finds exponentially fast depletion of states. The effective bounds on the depletion rates may depend on initial conditions.

SUPPLEMENTARY NOTE 3: LINEAR PROGRAMMING ALGORITHMS

In the following, we supplement the description and discussion of the linear programming algorithm from the Methods section in the main text. We detail on the CPEX algorithm and discuss its calibration, and provide a simplified Mathematica code.

CPLEX algorithm

The IBM ILOG CPLEX Optimization Studio 12.5 was used to numerically search for a condensate vector $\mathbf{c}$ for a given antisymmetric matrix A. Direct solution of the inequalities $A\mathbf{c} \leq 0$ and $\mathbf{c} - A\mathbf{c} \geq 1$ turned out to be numerically infeasible for systems with a large number of states S. Therefore, condensate vectors were primarily determined by solving the following linear programming problem: minimize $\epsilon_1 + \ldots + \epsilon_S$, subject to $-A\mathbf{c} + \epsilon \geq 0$ and $\mathbf{c} - A\mathbf{c} \geq 1$, with non-negative auxiliary variables $\epsilon := (\epsilon_1, \ldots, \epsilon_S)$. The resulting vector $\mathbf{c}$ was used to define the set $I := \{i \,|\, c_i > 5 \cdot 10^{-8}\}$ and its complement $\bar{I} = \{1, \ldots, S\} - I = \{i \,|\, c_i \leq 5 \cdot 10^{-8}\}$. The vector $\mathbf{c}$ was accepted as condensate vector, the set I as set of condensates, and the set $\bar{I}$ as set of depleted states if: $|(A\mathbf{c})_i| < 10^{-6}$ for all $i \in I$, and $-(A\mathbf{c})_i > 10^{-6}$ for all $i \in \bar{I}$. Minor extensions were added to the CPLEX algorithm to handle matrices for which an appropriate condensate vector could not be found. The Mathematica code in the following section exemplifies one of these extensions.

The above numerical thresholds were optimized by comparing inferred sets of condensates and of depleted states to sets that were derived using an alternative method. This alternative method is based on an analytical expression for kernel vectors (see equation (19) in the Methods section of the main text and [13, 14]). It is reliable but restricted to systems in which the network of states has connectivity $C = 1$ and in which the number of states is small (computational complexity grows exponentially with S). The correct identification of condensates and depleted states by the CPLEX algorithm was validated for 10^6 randomly sampled networks of states with connectivity $C = 1$ and $S = 18$ states. A detailed evaluation of the reliability of the CPLEX linear programming algorithm is provided in Supplementary Figs. 3 and 4.

Mathematica code

The following code for Mathematica 9.0 from Wolfram Research determines a condensate vector $\mathbf{c}$ by minimizing $\epsilon_1 + \ldots + \epsilon_S$, subject to $-A\mathbf{c} \geq 0$ and $\mathbf{c} - A\mathbf{c} + \epsilon \geq 1$. The resulting vector is used to infer the set of condensates I and the set of depleted states $\bar{I}$. The code can be used to verify the selection of states for the systems shown in Supplementary Figs. 1 and 2.

```
lpAlgorithm[noOfStates_, matrix_] :=
  Block[{condensateVector, condensates, depletedStates},
    Block[
    {
    solution
    (* solution: first half: condensate vector b, second half: aux. vector ε *),
    vector = Join[ConstantArray[0., noOfStates], ConstantArray[1., noOfStates]]
    (* vector: first half: w.r.t condensate vector b, second half: w.r.t. aux. vector ε *),
    lhs = ConstantArray[0., {2*noOfStates, 2*noOfStates}]
    (* lhs: upper left: -Ab, lower half: b-Ab+ε *),
    rhs = ConstantArray[0., 2*noOfStates]
    (* rhs: upper left: 0, lower half: 1 *)
    },

    Do[
      (* -Ab >= 0 *)
      Do[lhs[[i, j]] = -matrix[[i, j]], {j, 1, noOfStates}];
      rhs[[i]] = 0.;

      (* b-Ab+ε >= 1 *)
      lhs[[noOfStates + i, i]] = 1.;
      Do[lhs[[noOfStates + i, j]] -= matrix[[i, j]], {j, 1, noOfStates}];
      lhs[[noOfStates + i, noOfStates + i]] = 1.;
      rhs[[noOfStates + i]] = 1.;
    , {i, 1, noOfStates}];

    solution = Check[LinearProgramming[vector, lhs, rhs], {}];
    condensateVector = solution[[1;;noOfStates]];
    ];

  condensates = Flatten[Position[condensateVector, _?(# > 0.1&)]];
  depletedStates = Complement[Range[noOfStates], condensates];
  Return[{condensates, depletedStates, condensateVector/Total[condensateVector]}];
  ];
```

The following code can be used to verify the selection of states for the system with five states shown in Supplementary Fig. 2:

```
noOfStates = 5;
a12 = 8.3;
matrix = {{0,a12,4,-3,-5},{-a12,0,3,4,-5/3},{-4,-3,0,5,1},{3,-4,-5,0,5},{5,5/3,-1,-5,0}};

{condensates, depletedStates, condensateVector} = lpAlgorithm[noOfStates, matrix];

Print["Condensates: ", Length[condensates]];
Print["   Indices: ", condensates];
Print["   b: ", condensateVector[[condensates]]];
Print["   A.b: ", (matrix.condensateVector)[[condensates]]];
Print[];

Print["Depleted states: ", Length[depletedStates]];
Print["   Indices: ", depletedStates];
Print["   b: ", condensateVector[[depletedStates]]];
Print["   A.b: ", (matrix.condensateVector)[[depletedStates]]];
Print[];
```

To verify the selection of states for the system with 20 states that is shown in Supplementary Fig. 1, the first two lines of the above code have to be changed to:

```
noOfStates = 20;
matrix=
{{0.0000000000,0.1012965582,0.0960864501,0.1257833702,-0.0595764929,0.0924501179,-0.1016301739,
-0.0795618281,0.0487881199,-0.1122071087,-0.0286971727,-0.1000496191,-0.0103185636,0.0130714180,
-0.0572150115,-0.0129797149,-0.0706576396,-0.0389344063,0.0663966936,0.1467041892},{-0.1012965582,
0.0000000000,-0.0712011514,0.0415422138,-0.0019111200,-0.0816168047,-0.0477211059,-0.1006350936,
0.1522277516,0.0720296603,-0.2058646002,-0.1253355555,0.0717377587,-0.1063033227,-0.0942343547,
-0.0391532296,0.0049849208,-0.0929232005,-0.0840063075,0.0559612167},{-0.0960864501,0.0712011514,
0.0000000000,-0.0304803123,-0.1741821153,0.0076812872,0.0297497936,-0.0075143775,-0.0120359985,
-0.0110515825,-0.0808005342,0.2738320344,0.1170451029,-0.0357777508,-0.0896471841,-0.1577036387,
-0.3757310353,0.0296054422,-0.2259424452,0.0989073494},{-0.1257833702,-0.0415422138,0.0304803123,
0.0000000000,0.0510755453,0.0115416481,0.0985481106,0.0272202852,0.0213164598,-0.0535439544,
-0.0456534129,0.1362532620,0.1074372756,0.0375029473,-0.0533083982,0.1648089686,-0.0526653130,
0.0389051602,0.0438611333,0.0539352012},{0.0595764929,0.0019111200,0.1741821153,-0.0510755453,
0.0000000000,0.0109896214,0.1353304974,0.0912349277,0.0296142827,-0.1538255140,0.0431260101,
-0.0502994390,-0.0163964089,-0.1759377604,0.0481992186,0.0664062093,0.1736770927,-0.0448630119,
0.0535605474,-0.0257754999},{-0.0924501179,0.0816168047,-0.0076812872,-0.0115416481,-0.0109896214,
0.0000000000,-0.1090515383,-0.1188694334,0.0514000882,-0.0331443533,0.1623742129,0.0492738763,
0.0176787814,-0.1341072593,-0.0009543378,-0.0789222831,-0.0579314512,0.0892386351,-0.0686414708,
0.0492364011},{0.1016301739,0.0477211059,-0.0297497936,-0.0985481106,-0.1353304974,0.1090515383,
0.0000000000,-0.0303370160,0.0506580949,0.0225254369,0.1119589132,-0.2732763845,0.0903284019,
0.0780506743,0.1487517615,-0.0050831919,-0.0357202770,-0.1006725919,-0.0014275275,0.0744309213},
{0.0795618281,0.1006350936,0.0075143775,-0.0272202852,-0.0912349277,0.1188694334,0.0303370160,
0.0000000000,0.0881041025,0.0210453129,-0.0131581374,-0.0515644614,0.0300418276,0.0765770257,
0.1482013668,0.0876706565,-0.1600022303,-0.1501954314,0.0381309952,0.1069018065},{-0.0487881199,
-0.1522277516,0.0120359985,-0.0213164598,-0.0296142827,-0.0514000882,-0.0506580949,-0.0881041025,
0.0000000000,-0.0937867616,-0.1253362044,0.1051027292,-0.0160557361,0.0120747605,0.0410327424,
-0.1178120937,-0.0104974723,0.1001865178,0.0915443356,-0.0590317396},{0.1122071087,-0.0720296603,
0.0110515825,0.0535439544,0.1538255140,0.0331443533,-0.0225254369,-0.0210453129,0.0937867616,
0.0000000000,-0.1718419085,0.0842948432,0.1084407671,-0.1297238294,0.0768833880,-0.0866723482,
0.0062786219,-0.0986826408,-0.1352434973,-0.1425316892},{0.0286971727,0.2058646002,0.0808005342,
0.0456534129,-0.0431260101,-0.1623742129,-0.1119589132,0.0131581374,0.1253362044,0.1718419085,
0.0000000000,0.1936649126,-0.0428450371,0.0782428143,0.0592244942,-0.0554995964,0.0105651402,
-0.0184545141,0.1317087624,-0.1175718037},{0.1000496191,0.1253355555,-0.2738320344,-0.1362532620,
0.0502994390,-0.0492738763,0.2732763845,0.0515644614,-0.1051027292,-0.0842948432,-0.1936649126,
0.0000000000,0.0668839360,0.0174093226,0.0573713882,-0.0610992396,-0.0280347431,0.1058623608,
0.1781275929,0.0152443634},{0.0103185636,-0.0717377587,-0.1170451029,-0.1074372756,0.0163964089,
-0.0176787814,-0.0903284019,-0.0300418276,0.0160557361,-0.1084407671,0.0428450371,-0.0668839360,
0.0000000000,0.0571450290,0.1871354534,0.0147123474,0.1010276308,0.0366350187,0.0630761367,
-0.0728212225},{-0.0130714180,0.1063033227,0.0357777508,-0.0375029473,0.1759377604,0.1341072593,
-0.0780506743,-0.0765770257,-0.0120747605,0.1297238294,-0.0782428143,-0.0174093226,-0.0571450290,
0.0000000000,0.0158510770,-0.0301637492,0.0379895572,0.0353221008,-0.0410300505,0.0399902646},
{0.0572150115,0.0942343547,0.0896471841,0.0533083982,-0.0481992186,0.0009543378,-0.1487517615,
-0.1482013668,-0.0410327424,-0.0768833880,-0.0592244942,-0.0573713882,-0.1871354534,-0.0158510770,
0.0000000000,-0.1410043405,0.0473724443,0.1164556594,0.0120263929,0.0383652365},{0.0129797149,
0.0391532296,0.1577036387,-0.1648089686,-0.0664062093,0.0789222831,0.0050831919,-0.0876706565,
0.1178120937,0.0866723482,0.0554995964,0.0610992396,-0.0147123474,0.0301637492,0.1410043405,
0.0000000000,-0.0051486466,0.0770482371,-0.0619160030,0.1041763643},{0.0706576396,-0.0049849208,
0.3757310353,0.0526653130,-0.1736770927,0.0579314512,0.0357202770,0.1600022303,0.0104974723,
-0.0062786219,-0.0105651402,0.0280347431,-0.1010276308,-0.0379895572,-0.0473724443,0.0051486466,
0.0000000000,0.0771683333,0.0494219197,0.3405228485},{0.0389344063,0.0929232005,-0.0296054422,
-0.0389051602,0.0448630119,-0.0892386351,0.1006725919,0.1501954314,-0.1001865178,0.0986826408,
0.0184545141,-0.1058623608,-0.0366350187,-0.0353221008,-0.1164556594,-0.0770482371,-0.0771683333,
0.0000000000,-0.1856676553,0.0357968997},{-0.0663966936,0.0840063075,0.2259424452,-0.0438611333,
-0.0535605474,0.0686414708,0.0014275275,-0.0381309952,-0.0915443356,0.1352434973,-0.1317087624,
-0.1781275929,-0.0630761367,0.0410300505,-0.0120263929,0.0619160030,-0.0494219197,0.1856676553,
0.0000000000,0.1384008887},{-0.1467041892,-0.0559612167,-0.0989073494,-0.0539352012,0.0257754999,
-0.0492364011,-0.0744309213,-0.1069018065,0.0590317396,0.1425316892,0.1175718037,-0.0152443634,
0.0728212225,-0.0399902646,-0.0383652365,-0.1041763643,-0.3405228485,-0.0357968997,-0.1384008887,
0.0000000000}};
```

SUPPLEMENTARY REFERENCES

[1] M. A. Nowak and K. Sigmund, Science **303**, 793 (2004).

[2] D. Vorberg, W. Wustmann, R. Ketzmerick, and A. Eckardt, Phys. Rev. Lett. **111**, 240405 (2013).

[3] M. Grifoni and P. Hänggi, Phys. Rep. **304**, 229 (1998).

[4] C. W. Gardiner and P. Zoller, *Quantum Noise* (Springer, Berlin Heidelberg, 2004).

[5] H.-P. Breuer and F. Petruccione, *The Theory of Open Quantum Systems* (Oxford University Press, Oxford, 2002).

[6] C. Gardiner, *Stochastic Methods: A Handbook for the Natural and Social Sciences* (Springer, Berlin, 2009).

[7] U. C. Täuber, *Critical Dynamics* (Cambridge University Press, Cambridge, MA, 2014).

[8] J. Hofbauer and K. Sigmund, *Evolutionary Games and Population Dynamics* (Cambridge University Press, Cambridge, 1998).

[9] N. G. Van Kampen, *Stochastic Process in Physics and Chemistry* (Elsevier, Amsterdam, 2007).

[10] H. Kuhn and A. Tucker, *Linear Inequalities and Related Systems* (Princeton University Press, Princeton, NJ, 1956).

[11] R. M. May, *Stability and Complexity in Model Ecosystems* (Princeton University Press, Princeton, NJ, 1973).

[12] S. Wiggins, *Introduction to Applied Nonlinear Dynamical Systems and Chaos* (Springer-Verlag, New York, 2003).

[13] C. E. Cullis, *Matrices and Determinoids*, Vol. I and II (Cambridge University Press, Cambridge, 1913).

[14] J. Knebel, T. Krüger, M. F. Weber, and E. Frey, Phys. Rev. Lett. **110**, 168106 (2013).
